# Reversal of proliferation deficits caused by chromosome 16p13.11 microduplication through targeting NFκB signaling: an integrated study of patient-derived neuronal precursor cells, cerebral organoids and in vivo brain imaging

**DOI:** 10.1038/s41380-018-0292-1

**Published:** 2018-11-06

**Authors:** Mandy Johnstone, Navneet A. Vasistha, Miruna C. Barbu, Owen Dando, Karen Burr, Edward Christopher, Sophie Glen, Christelle Robert, Rana Fetit, Kenneth G. Macleod, Matthew R. Livesey, David St. Clair, Douglas H. R. Blackwood, Kirsty Millar, Neil O. Carragher, Giles E. Hardingham, David J. A. Wyllie, Eve C. Johnstone, Heather C. Whalley, Andrew M. McIntosh, Stephen M. Lawrie, Siddharthan Chandran

**Affiliations:** 1 Division of Psychiatry, University of Edinburgh, Royal Edinburgh Hospital, Edinburgh, UK; 2 Centre for Genomic and Experimental Medicine, MRC Institute of Genetics and Molecular Medicine, University of Edinburgh, Edinburgh, UK; 3 Centre for Clinical Brain Sciences, University of Edinburgh, Edinburgh, UK; 4 UK Dementia Research Institute at University of Edinburgh, Edinburgh Medical School, Edinburgh, UK; 5 Centre for Discovery Brain Sciences, University of Edinburgh, Hugh Robson Building, 15 George Square, Edinburgh, UK; 6 Royal (Dick) School of Veterinary Studies, The Roslin Institute, University of Edinburgh, Edinburgh, UK; 7 Cancer Research UK Edinburgh Centre, MRC Institute of Genetics and Molecular Medicine, University of Edinburgh, Edinburgh, UK; 8 Institute of Medical Sciences, University of Aberdeen, Foresterhill, Aberdeen, UK; 9 Centre for Brain Development and Repair, Bangalore, India

**Keywords:** Stem cells, Schizophrenia, Cell biology, Biomarkers

## Abstract

The molecular basis of how chromosome 16p13.11 microduplication leads to major psychiatric disorders is unknown. Here we have undertaken brain imaging of patients carrying microduplications in chromosome 16p13.11 and unaffected family controls, in parallel with iPS cell-derived cerebral organoid studies of the same patients. Patient MRI revealed reduced cortical volume, and corresponding iPSC studies showed neural precursor cell (NPC) proliferation abnormalities and reduced organoid size, with the NPCs therein displaying altered planes of cell division. Transcriptomic analyses of NPCs uncovered a deficit in the NFκB p65 pathway, confirmed by proteomics. Moreover, both pharmacological and genetic correction of this deficit rescued the proliferation abnormality. Thus, chromosome 16p13.11 microduplication disturbs the normal programme of NPC proliferation to reduce cortical thickness due to a correctable deficit in the NFκB signalling pathway. This is the first study demonstrating a biologically relevant, potentially ameliorable, signalling pathway underlying chromosome 16p13.11 microduplication syndrome in patient-derived neuronal precursor cells.

## Introduction

Species-specific evolutionary expansion of cortical structure and circuitry underlies the unique range of human cognitive abilities. Whilst rodent models of brain development and function are invaluable, inherent differences in the organisation and structure of the human neocortex requires complementary human experimental systems to model aspects of neurodevelopmental disorders such as schizophrenia (SCZ) and autism spectrum disorders (ASDs). Human induced-pluripotent stem cell (iPSC) technology, coupled to genetic discoveries, and the ability to reconstruct 3D cortical structures offers an unprecedented opportunity to better understand the mechanisms underlying major psychiatric disorders.

Copy number variations (CNVs), such as those found at chromosome 16p13.11 locus are associated with a range of phenotypically different neurodevelopmental disorders including SCZ [1–4], intellectual disability [5, 6] and ASD [5], which suggest that the locus contains dosage-sensitive gene(s) with a critical role in neurodevelopment. The deCODE genetics study of 4345 patients with SCZ and 35,079 controls from eight European populations found a threefold excess of duplications and deletions at the 16p13.1 locus in SCZ cases, with duplications being far more commonly found [3]. Mirroring this, in a Scottish population sample we found a fourfold excess of duplications at the 16p13.1 locus in SCZ patients compared to controls [7].

Despite this, the molecular basis of how 16p13.11 microduplication leads to major psychiatric disorders is unknown. Ingason et al. [3] subdivided the 16p13.11 region, between 14.66 and 18.70 Mb, into three single-copy sequence intervals, denoted intervals I, II and III, each of which is flanked by sequences rich in low copy repeats (LCR). All duplications and deletions so far reported are contained within this region with the most common breakpoints in the LCR clusters distal to interval I and proximal to interval II (so called Dup I + II carriers) [3]. Given that Dup I + II carriers showed the highest common odds ratio of all 16p13.11 microduplication carriers, we decided to focus on familial carriers of this CNV. The core genetic locus, conserved across mice and humans, contains seven genes and it has long been proposed that a key gene in this region is *NDE1* (*Nuclear Distribution protein nudE homolog 1 also known as nude neurodevelopmental protein*) with deletions and duplications spanning *NDE1* amongst the most common CNVs in SCZ [3]. However, a recently described bacterial artificial chromosome-transgenic mouse model, carrying a human 16p13.11 locus, has been shown to exhibit a behavioural hyperactivity phenotype associated with miR-484 overexpression with resultant decreased proliferation and increased differentiation of neural progenitors in vivo [8] calling into question whether the key gene is *NDE1* or another gene in the locus such as miR-484 or indeed a combination of these genes that is responsible for the phenotypes associated with the 16p13.11 microduplication.

Over the years the evidence for *NDE1* has mounted from rodent studies: *NDE1* encodes a cytoskeletal protein that is part of the LIS1/cytoplasmic dynein complex localising to the centrosome [9–11] where it participates in a range of essential neurodevelopmental processes including NPC proliferation, neuronal migration, intracellular transport and neurite outgrowth [12–14]. NDE1 and its paralogue NDEL1 (*Nuclear distribution protein nudE-like 1*) form an epistatic interaction where NDE1 and NDEL1 compete with one another for binding to the SCZ risk factor DISC1 [15] with mutations in both *NDE1* and *NDEL1* resulting in defective neurogenesis and neuronal migration [16–19]. Recently, it has been shown that DISC1 regulates neurogenesis via modulating kinetochore attachment of NDEL1/NDE1 during mitosis, demonstrating a critical role of the DISC1/NDEL1 interaction in regulating mitosis of radial glial progenitors (RGPs), both in the developing mouse cortex *in vivo* and in human forebrain organoids from an individual with schizophrenia who carries a 4-bp deletion of *DISC1* [20]. This mutation results in the production of truncated DISC1, abolishing its interaction with NDEL1. Furthermore, it has been shown that familial mutations in *NDE1* can cause both severe failure of neurogenesis and a deficiency of cortical lamination [21, 22].

Rare single-nucleotide polymorphisms (SNPs) in *NDE1* also associate with SCZ and psychosis susceptibility [23, 24], with the latter conditioned on a *DISC1* associating risk haplotype [24]. Taken together, the genetic and biological evidence for DISC1 and NDE1 suggest a shared ‘risk’ pathway in neurodevelopmental disorders although the precise mechanism(s) of action has remained elusive. Recently, Bradshaw et al. [25], using gene expression and psychoactive medication data, have elegantly demonstrated that the *NDE1* SNP rs2242549 associates with significant changes in gene expression and that a significant number of these were predicted targets of miR-484, located within a non-coding exon of *NDE1*. This has led to the hypothesis that variation at the *NDE1* locus may alter risk of mental illness, in part through modification of miR-484, highlighting this as a potentially important locus in targeting treatment [25].

We hypothesized that genetic interactions, within the biological network linked to specific genes within 16p13.11 dup, would rise to the level of clinical association and that patient-derived iPSC studies could provide an important opportunity to identify these pathways. Structural brain imaging of carriers compared to unaffected family controls revealed reduced cortical volumes that was mirrored by smaller cerebral organoids in vitro. Furthermore, we show reduced ventricular zone and NPC proliferation associated with deficits in the NF*κ*B p65 pathway that upon pharmacological or genetic activation corrects the proliferation deficit. This study integrates patient and cellular studies to demonstrate a role for genes within the 16p13.11 locus (in particular *NDE1*) in human corticogenesis, and implicates dysregulation of NF*κ*B as an important potential mechanistic pathway in neurodevelopmental and neuropsychiatric disorders.

## Materials and methods

### Patient recruitment and assessment

Patient diagnosis was carried out, according to ICD-10 and DSM-IV-TR criteria. The study was approved by the Multicentre Research Ethics Committee for Scotland (REC reference: 13/SS/0039; IRAS project ID: 147086). A detailed description of the study was given and written informed consent was obtained from all individuals prior to participation. A summary of the individual participants, their diagnosis, CNV status, medications at the time of study, and their age at onset, biopsy and MRI is shown in Supplementary Table 1 and detailed in supplementary methods.

### Structural MRI data acquisition

**Fig. 1:**
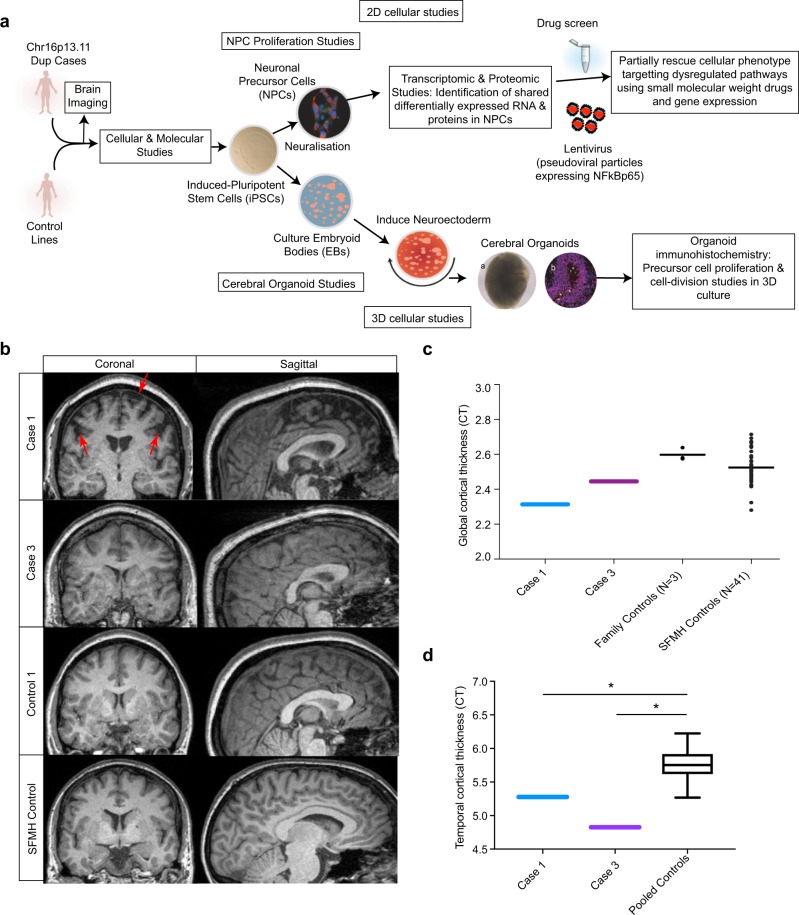
Overview of the study design and MR imaging shows cortical deficits in carriers of the chr16p13.11 duplication. **a** Schematic summary of the study. **b** Coronal and sagittal MRI views demonstrate significant volume reduction of cortical brain tissue in an affected carrier of the 16p13.11 microduplication (case 1) evident in both the coronal (areas of significant cortical volume reduction are marked by red arrows) and sagittal views. Representative scans are also shown from an in-family control (control 1) and from an age and gender matched participant in the Scottish Mental Health Research study (SFMH) control group. Representative MRI images are also shown for case 3. Case 2 was unable to tolerate the MR scan due to extreme anxiety and agitation. **c** Global cortical thickness measures show significant differences between case 1 and a group of 41 control individuals from the SMHR study. Significance testing on the differences between case 1’s score and the SFMH control sample are as follows based on N-of-1 statistics [67]: control sample *t* = −2.305; one-tailed probability = 0.013; two-tailed probability = 0.026. Estimated percentage of normal population falling below individual's score = 1.32%. 95% lower confidence limit on the percentage = 0.17%. 95% upper confidence limit on the percentage = 4.14%. The value for mean global CT is also marked in this graph for case 3. **d** Temporal lobe cortical thickness measures show significant differences between cases 1 and 3 and a group of 41 control individuals from the SMHR study

All brain images were acquired on a Siemens Skyra 3.0T scanner (Siemens Medical Solutions, Germany) (see supplementary materials). As NDE1 is hypothesised to have an important role in neuronal proliferation, migration and corticogenesis, we focused structural imaging analysis on measures of cortical thickness (CT). FreeSurfer software (v5.3) [26] was used to derive measures of global and lobar cortical thickness (frontal, temporal, parietal and occipital). Formal N-of-1 statistical tests were performed in order to test CT differences between each of the affected carriers with imaging data (case 1 and case 3; case 2 was unable to tolerate MRI) against 41 non-relative, unaffected, non-carriers within the Scottish Mental Healthy Family (SFMH) who were used as the normative comparison group. To remove any confounding effects of age or gender on CT values, both groups were matched as closely as possible (cases versus control; mean age = 35.5 versus 38.3 years; %Female:Male ratio = 50:50 versus 44:56, respectively). False discovery rate (FDR) correction for multiple comparisons was applied across measures of CT for each comparison. For direct comparison to the most severely affected carrier (case 1), we have shown a representative MRI scan of an unaffected, non-carrier participant within the SFMH study, who has been matched for age (47 and 45 years of age, respectively) and gender (both female) (Fig. 1b). We also included MRI scans from three healthy unaffected non-carrier relatives from within the 16p13.11 duplication families and these do not show any deficits in cortical thickness (Fig. 1c).

### Reprogramming fibroblasts to generate iPSC and three germ layer differentiation

Human iPSC were derived from donor skin fibroblasts using integration-free episomal methods [27] (see supplementary methods). Control 5 was generated by retrovirally reprogramming fibroblasts [28]. To validate differentiation capacity of iPSCs, we performed in vitro differentiation into three germ layers: endoderm, mesoderm, neuroectoderm as detailed in supplementary methods.

### NPC proliferation assays: EdU labeling and detection

Anterior NPCs (aNPCs) were derived from parental iPSC lines as detailed in supplementary methods. Following plate down in RosV2 F10 [Advanced-DMEM/F12, 1% P/S, 1% Glutamax, 1% N-2, 0.5% B-27, supplemented with 2.5 ng/mL FGF-2] media was changed to default [Advanced-DMEM/F12, 1% P/S, 0.5% Glutamax, 0.5% N-2, 0.2% B-27, 2 μg/mL Heparin (Sigma)] to remove the effects of mitogens. To assess NPC proliferation mitotic cells were grown to ~50% confluency (to ensure consistency in experimental conditions) and labelled with 10 μM ethynyl deoxy-uridine (Click-IT EdU, Life Technologies) for 4 h. Media was replaced completely and cells cultured for an additional 4 h in the absence of EdU before fixation using 4% paraformaldehyde (PFA) (Sigma). Cells were permeabilised using 0.5% Triton X-100 (Sigma) in PBS for 20 mins and detected using the EdU detection kit (ThermoFisher) according to manufacturer’s instructions. NPCs were also immunolabelled for Nestin and Pax-6 (see Supplementary Table 2 for antibody details). Coverslips were washed and mounted using ProLong Gold antifade mountant with 4′,6-Diamidine-2′-phenylindole dihydrochloride (DAPI) (ThermoFisher).

### Cerebral organoid experiments

Cerebral organoids were generated and maintained according to published protocols [29, 30] with the following minor modifications. hiPSCs were cultured in Matrigel-coated plates in E8 medium (Life Technologies). When confluent, they were lifted into a 6 well plate in Phase I medium using dispase/collagenase treatment. They were grown in 10 cm dishes containing Phase I medium for 7 days and then changed to EB1 medium [A-DMEM/F12**;** 1% PSF**;** 1% Glutamax**;** 1% N-2**;** 500 μL B-27**;** 12.5 μL FGF-2] for a further 2–4 days depending on the nature of cells. After onset of rosette patterning embryoid bodies (EBs) were collected in a 15 mL falcon tube, media was removed and replaced with EB2 medium [same as EB1 except it contains B-27 without vitamin A and NEAA] in a fresh 10 cm dish. Thereafter, EBs were cultured in EB3 media (Same as EB2, except contains B-27 + Vitamin A and 1% Matrigel) which was changed every second day. Cultures were grown on a digital orbital shaker (Heathrow Scientific). Tissue was processed and analysed as described in supplementary methods.

### RNA sequencing

RNAs were isolated using the RNeasy Mini kit (QIAGEN). RNA-seq samples were prepared from 12 NPC lines [8 control and 4 case lines comprising: control 1 (2 iPSC clones, from which one NPC line was derived from clone 1 and 2 NPC lines from clone 2, i.e. a total of 3 RNA samples were sequenced from control 1); control 2 (1 iPSC clone was derived into 1 NPC line from which 1 RNA-seq sample was prepared); control 3 (1 iPSC clone from which 2 NPC lines were derived providing 2 RNA-seq samples); control 4 (1 iPSC clone from which 1 NPC line was derived providing 1 RNA-seq sample); control 5 (1 iPSC clone from which 1 NPC line was derived providing 1 RNA-seq sample); case 1 (3 iPSC clones were generated and derived to NPCs from which 2 were used for RNA-seq); case 2 (2 iPSC clones were generated and derived to NPCs from which 1 was used for RNA-seq); case 3 (2 iPSC clones were generated and derived to NPCs from which 1 was used for RNA-seq); see Supplementary Table 3 for the specific lines used]. Genomic DNA was removed with the DNA-Free kit (Ambion). Analysis was performed as described in supplementary methods.

### Reverse-phase protein arrays (RPPAs)

RPPA is a miniaturised, highly sensitive, quantitative “dot-blot” immunoassay where the analyte (from cell lysates) is immobilised on a solid phase (nitrocellulose) and subsequently probed with antibodies to specific targets (Fig. 4a) [31, 32] ideally suited to elucidate mechanisms-of-action and phenotypic response in 2D and 3D models [33]. Forty candidate intracellular signalling molecules were examined (see Supplementary Table 4). The specific lines used in RPPA experiments are detailed in Supplementary Table 3. Samples were prepared as described in supplementary methods.

### Screening of small-molecule activators of NFκB p65

Two small molecular weight activators of NF*κ*B p65 (SRI-22772 and SRI-22782; denoted Compounds 1 and 2, respectively) previously shown to result in non-canonical activation of NF*κ*B p65 in neurons [34] (ChemBridge Corporation, San Diego, California) were solubilised in 100% DMSO (Sigma) at a concentration of 5 μg/mL. As a positive control NPCs were treated with TNF-α (Peprotech) at 100 ng/mL.

### Transient transfection of NPCs with NDE1

pcDNA3.1NDE1-WT-V5 construct was generated by amplifying cDNA fragments encoding C-terminally V5-tagged wild-type human NDE1 using pDEST40 NDE1 constructs as templates, digested with *Bam*HI/*Not*I restriction enzymes then inserted into the *Bam*HI/*Not*I site of pcDNA3.1 (ThermoFisher) as described previously [35]. NPCs were transiently transfected using an Amaxa Nucleofector device (Lonza). Briefly, 5 × 10^6^ cells were lifted with accutase, pelleted by centrifugation (200×*g* for 5 min), washed with PBS and resuspended in 82 μL of Amaxa nucleofection solution with 18 μL of Amaxa nucleofection supplement 1. Cell suspensions were transferred to 1.5 mL Eppendorf tubes with 5–10 μg of plasmid DNA (either pcDNA3.1NDE1-WT-V5 or pcDNA3.1 plasmid alone control). The cell–DNA mix was transferred to an Amaxa cuvette, and electroporation was carried out using programme “A-033”. Cells were then plated in default media on poly-ornithine (Sigma), laminin (Sigma), fibronectin (Sigma) and Matrigel-coated coverslips and left for 48 h to recover post-transfection. NPC proliferation assays were then performed using the Click-IT EdU kit (ThermoFisher) as described above but left for an extended period of 10 h. Cells were fixed and stained as described or lifted with accutase, washed and centrifuged for preparation of cell lysates for immunoblotting as described in supplementary methods.

### Lentivirus treatment of NPCs

A total of 100 μL of lentivirus (production detailed in supplementary methods) was mixed with 4 μg/mL of polybrene (Merck) and used to infect 1 × 10^6^ NPCs grown in 2 mL of media. The cells were incubated with the virus for 6 h at 37 °C. Thereafter, the virus containing media was disposed and replaced with fresh media and NPCs were left for 48 h. Cells were then processed for cell proliferation assays as described above or lifted with accutase, centrifuged, washed in PBS and cell pellets were processed for immunoblotting.

## Results

### Reduced cortical thickness in chromosome 16p13.11 microduplication carriers

To study the impact of 16p13.11 microduplications on cortical thickness in humans, we undertook structural MRI scanning alongside human stem cell studies (Fig. 1). Structural MRI scans show marked cortical deficits in the coronal and sagittal planes, particularly in the frontal lobe compared to controls (Fig. 1b–d). Case 1 demonstrated significantly reduced CT globally, and in frontal and parietal lobar regions versus controls (*t* = −2.31, *p* = 0.01, *t* = − 1.88, *p* = 0.03, *p*_corr_ = 0.04, *t* = −2.06, *p* = 0.02, *p*_corr_ = 0.04, global, frontal and parietal, respectively, Fig. 1c). Both case 1 and case 3 demonstrated significantly reduced CT in the temporal lobe versus the healthy comparison subjects (Fig. 1d) (case 1: *t* = −2.13, *p* = 0.02, *p*_corr_ = 0.04; case 3: *t* = −4.04, *p* = 0.00, *p*_corr_ < 0.01).

### Proliferation deficits in case-derived NPCs

To determine the cellular and molecular basis underlying the observed abnormalities in cortical volume, we next established multiple iPSC lines, using episomal methods, from three affected individuals (from two families previously identified [7]) carrying 16p13.11 microduplications in one chromosome (cases 1–3) and from five control individuals, including two from the same family as cases 2 and 3 (Supplementary Table 1 and Supplementary Figure 1b and c). All iPSCs were karyotypically normal, expressed pluripotency markers and showed three germ layer differentiation (Supplementary Figure 1a and 2). Long-term propagated monolayer cultures of anterior NPCs displayed rosette-like patterns with typical NESTIN and OTX2 expression (Supplementary Figure 3a-b). When aNPCs were differentiated by plate down in default media supplemented with forskolin, BDNF and GDNF (Supplementary Figure 3c-d), immunocytochemistry at 35 days revealed predominantly neuronal cultures (assessed by MAP2 staining) with <15% astrocyte (assessed by GFAP staining)(Supplementary Figure 3e).

**Fig. 2:**
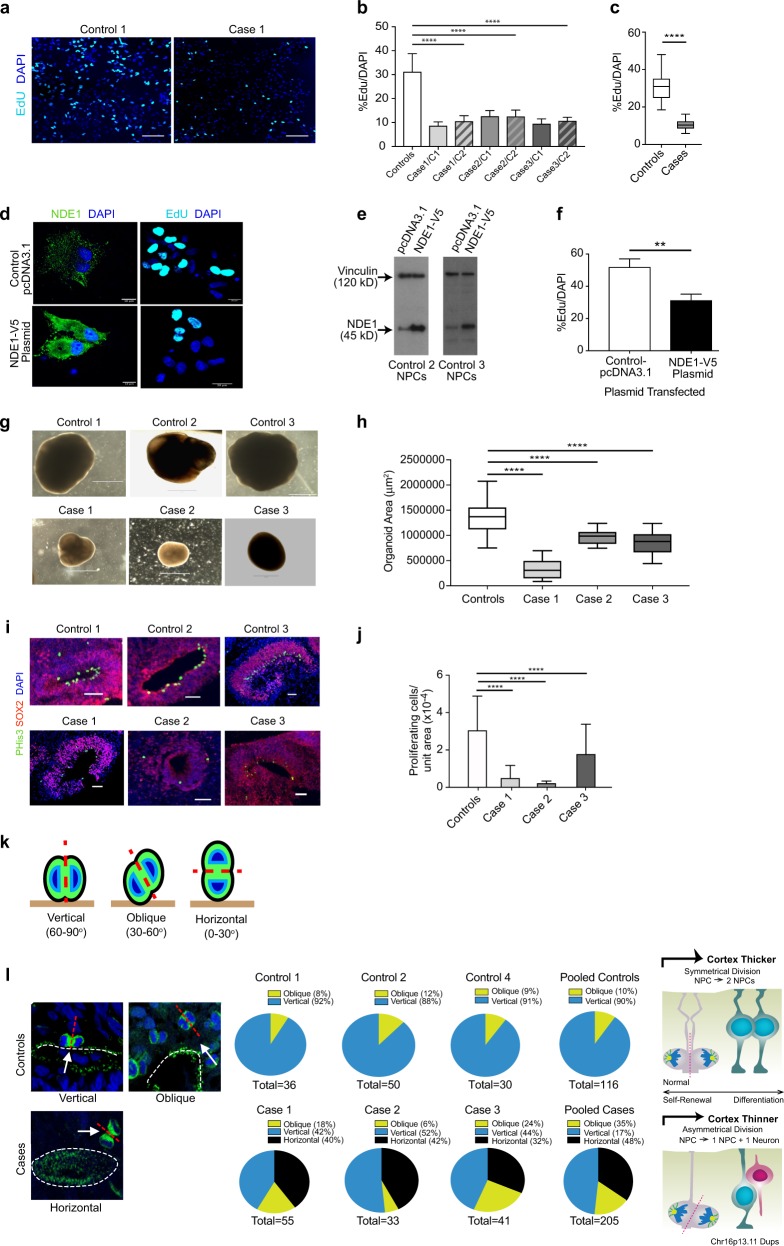
Proliferation studies of NPCs and cerebral organoids. **a** Representative images of the mean proliferation values of case 1 compared to control 1. Scale bar: 50 µm. NPCs from case and control lines were plated down and grown in default media for 7–10 days prior to Click-IT EdU proliferation assays being undertaken. **b** NPCs from affected cases carrying the Chr16p13.11 microduplication show significantly reduced proliferation compared to control lines. Two NPC lines derived from two independent iPSC clones from each case were compared to a panel of NPC lines derived from five control individuals. **c** Graph showing values for pooled control NPC lines compared to pooled case NPC lines. Graphs shows mean  ± sem, controls: 31.26 ± 1.14%, cases 10.5 ± 0.298%, ****p < 0.0001 (unpaired two-tailed *t*-test with Welch’s correction). **d** NPCs from control individuals (control 2 and 3) were transfected either with full-length human V5-tagged NDE1 (pcDNA3.1NDE1-WT-V5 construct) or with the plasmid alone (pcDNA3.1). NDE1 staining shows intense signal of NDE1 in the cytoplasm of cells which had been transfected with the *NDE1*-V5 construct compared to NPCs transfected with control plasmid. **e** Immunoblotting shows that NPCs, derived from healthy controls and transfected with pcDNA *NDE1*-WT-V5 construct, expressed significantly more NDE1 compared to NPCs transfected with pcDNA3.1-control plasmid alone. **f **Proliferation assays post-transfection: Electroporated cells were plated down and 48 h later EdU was added to measure the proliferation rate of transfected cells (right hand panel of **d**). **f** Graph shows that cells transfected with full-length wild-type human *NDE1* display significantly reduced proliferation over a 10-h time-period compared to empty vector transfected cells. Graph shows mean  ± sem, **p < 0.005 (*p* = 0.0036, paired *t*-test; mean = 51.98% EdU/DAPI for empty vector control transfected NPCs compared to mean = 31.3% EdU/DAPI for NPCs transfected with full-length *NDE1*). **g** Representative images of cerebral organoids from controls and cases at 1 month of age. Scale bar: 1000 μm (1 mm). **g**  Plot of organoid sizes (organoid area μm^2^) from controls and cases at 1 month. Data represented as box plots with central bar representing mean  ± sem shown by whiskers; ****p < 0.0001, ordinary one-way ANOVA with Dunnett’s multiple correction test. Case 1 organoids are significantly smaller than controls (mean area of 326,550 μm^2^ for case 1 organoids compared to mean of 1,364,157 μm^2^ for controls. Case 2 and 3 organoids were less severely stunted in their growth rates with mean areas of 973,586 and 856,846 μm^2^, respectively). Organoid *n* = 30 + per case. **i** Representative images of proliferative zones of 1-month-old organoids from control compared to case iPSC lines. Scale bar: 50 µm. **j **Graph of proliferating cells (P-His+ve) quantified in sectioned organoids showing pooled values of different clones for each case. Organoids were sectioned, stained and quantified using Image J by two independent blinded examiners. Family control organoids are from *n* = 3 individuals. Data represented as mean  ± sem; ****p < 0.0001, ordinary one-way ANOVA with Dunnett’s multiple correction test. Chr16p13.11 microduplications result in a switch from symmetrical to asymmetrical cell division. **k** Schematic diagram illustrating RG division in one of three orientations: 60–90 degrees (vertical); 30–60 degrees (oblique) and 0–30 degrees (horizontal). **l** Cerebral organoids were sectioned and stained with antibodies to phospho-histone H3 and/or phospho-vimentin. Quantification of RG division orientations is displayed in pie charts next to representative images for case and control organoids examined showing the proportion of cells in one of three bins (vertical, oblique or horizontal) *n* = 30–50 anaphase cells from a minimum of five cerebral organoids from each line. Schematic diagram (right) illustrating potential consequences to cortical thickness following a switch from symmetric to asymmetric cell division in affected carriers of Chr16p13.11 microduplications

In view of evidence that mutations in genes, particularly *NDE1*, in the microduplication are associated with abnormal neurogenesis and cortical lamination, we determined levels of NDE1 in NPCs [9, 19] (Supplementary Figure 4). This showed that patient iPSC-derived NPCs carry (i) three copies of *NDE1* at the genomic level (by digital-drop PCR), (ii) approximately 1.5× mRNA (by RT-qPCR) and (iii) 1.5× NDE1 protein by immunoblotting (Supplementary Figure 4b-d). Our RT-qPCR experiments also showed that several other genes within the chromosome 16p13.11 microduplication, in particular *NTAN1*, *PDXDC1* and *ABCC1* were also ~1.5× elevated at the mRNA level in cases compared to controls. We next compared proliferation of early passage (6–12) NPCs between cases and controls following plating in the absence of mitogens. Significantly reduced NPC proliferation, measured by Edu Click-It assays, was found in all cases when compared individually (Fig. 2a, b) or as pooled data to control NPCs (Fig. 2c). Case 1 appeared to exhibit the most pronounced proliferation deficits compared to controls mirroring the severity of the changes seen in the MRI of case 1.

To determine whether these observed proliferation deficits in cases were dependent on NDE1 levels, we transiently transfected V5-tagged full-length human *NDE1* into NPCs from controls 2 and 3 (Fig. 2d). Importantly, the miR-484 sequence, encoded within the 5′UTR of the *NDE1* gene itself, is not included in this construct as the *NDE1* gene was sub-cloned using a 5′ amplification primer that was downstream of the miR-484 yet immediately upstream of the start codon of *NDE1* [36]. NDE1 was widely distributed throughout the cytoplasm of NPCs in a punctate pattern (Fig. 2d). Successful transfection was also confirmed by quantitative immunoblotting (Fig. 2e). Proliferation assays revealed control lines 2 and 3, overexpressing full-length human *NDE1*, showed significantly less proliferation (31.3% EdU/DAPI for NDE1 transfected NPCs compared to 51.98% for controls, *p* = 0.0036, paired *t*-test) (Fig. 2f). Together these findings suggest that reduced NPC proliferation in control NPCs are most likely due to the overexpression of *NDE1* and not the miR-484.

### Smaller cerebral organoids display altered planes of cell division of radial glial progenitors in 16p13.11 microduplication carriers

In view of the twin observations of global cortical thinning in patient MRI scans and reduced NPC proliferation in patient-derived 2D cultures, we next grew 3D cerebral organoids (Fig. 2g). Cerebral organoids formed large continuous neuroepithelia surrounding a fluid-filled cavity reminiscent of a ventricle with characteristic apical staining of the neural specific N-cadherin (Supplementary Figure 5a). Immunohistological analysis revealed upregulation of the anterior brain markers FOXG1 and PAX6 indicating successful neural induction (Supplementary Figure 5b-c). Significantly smaller organoids were observed at 1 month from cases, especially case 1, compared to controls (Fig. 2h; *p* < 0.0001, ordinary one-way ANOVA with Dunnett’s multiple correction test), reminiscent of the reduced brain size seen in the patients, especially case 1 (Fig. 1b), and the reduced proliferation of NPCs in cases compared to controls, with case 1 again showing the most pronounced deficit (Fig. 2a–c). Immunohistochemical staining of progenitors (Fig. 2i) and neurons (Supplementary Figure 5d) at 1 month also revealed far fewer neuronal progenitor cell regions and reduced numbers of total dividing neuronal progenitor cells in cerebral organoids as assessed by quantitative phospho-histone H3 (Ser10) and phospho-vimentin staining in all three affected carriers compared to controls (Fig. 2j; 84% reduction in proliferating cells per unit area of organoid from case 1; 93% reduction in case 2 and 40% reduction in case 3 compared to control-derived organoids; *p* < 0.0001, ordinary one-way ANOVA with Dunnett’s multiple correction test). Importantly, we were able to demonstrate recapitulation of cortical organization revealing typical arrangement into a layer reminiscent of the ventricular zone (VZ) with neurons located on the basal surface (Supplementary Figure 5d). Mitotic radial glia staining (phospho-histone H3) in cortical regions of 1-month-old cerebral organoids reveals ventricular radial glia (vRG) undergoing mitosis at the apical membrane whereas outer radial glia (oRG) undergo mitosis outside the ventricular zone. Radial glia in the ventricular zone are marked by SOX2 staining (Supplementary Figure 5d). Approximately 85% of P-His+ve cells are vRG cells and 15% are oRGs (±5.8%, *n* = 30).

RGPs constitute the majority of stem cells in the human neocortex and are highly elongated cells with an apical process in contact with the ventricular surface and a basal process in contact with the outer surface of the brain during development. The plane of division of RGPs is known to determine the fate of the daughter cells [37, 38]. We examined spindle-orientation in organoids by staining for phospho-histone H3 or phospho-vimentin, and classified the progenitors as having one of three positions relative to the ventricular surface: vertical, oblique or horizontal [37] (Fig. 2k). Control organoid tissue examined at 1 month of age showed exclusively vertical (60–90 degrees) and oblique (30–60 degrees) orientations. No horizontal orientations were observed in control organoids (Fig. 2l). In contrast, up to 20–42% of vRG were in a horizontal orientation (0–30 degrees) in case-derived lines. This switch to horizontal orientations may explain the patient phenotype of reduced cortical volume on MRI and reduced organoid size as symmetrical vRG division is important for progenitor self-renewal and determines cortical thickness formation during development [39]. Asymmetrical division on the other hand causes premature differentiation of one of the daughter cells to a neuronal fate thereby reducing the pool of progenitor cells and resulting in cortical thinning (schematic figure in Fig. 2l).

### Transcriptomic and proteomic analysis of case-derived NPCs and cerebral organoids identifies perturbations in the NFκB p65 pathway

**Fig. 3:**
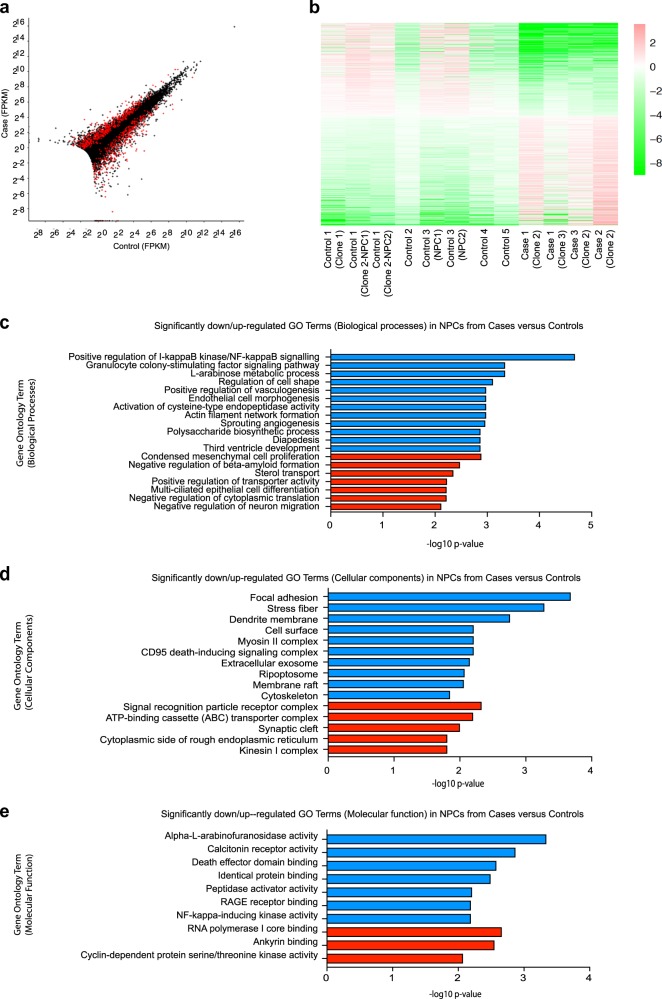
RNA-sequencing data highlights differences in I-kappaB kinase and NF-kappaB signalling pathways as well as focal adhesion, regulation of cell shape, actin filament network, cytoskeleton, and alpha-l-arabinofuranosidase activity. **a** Representation of the differential expression results. Each cross is a gene, plotted with its mean abundance (measured in “fragments per kilobase per million mapped reads” (FPKM)) in cases versus controls. Very low abundance genes (those with mean FPKM across all samples < 0.5) have been filtered out. Red crosses are those genes that were called as differentially expressed. Genes with zero mean FPKM are drawn superimposed on the axes. **b** Heatmap of differentially expressed genes. Colours indicate log2(gene abundance in sample/mean abundance for this gene). Ordering is from most downregulated in cases to most upregulated. Graphs showing the most significantly downregulated (blue) or upregulated (red) gene ontology (GO) terms for (**c**) biological processes, (**d**) cellular components and (**e**) molecular function

To explore potential molecular mechanisms underlying reduced NPC proliferation, we next undertook RNA-sequencing analysis (Fig. 3). Applying a FDR of 0.05, there are 994 differentially expressed genes between pooled case and control NPCs. Expression fold changes of the top 20 most significant up and downregulated genes are shown (Supplementary Figure 6a) and all differentially expressed genes are listed in Supplementary Table 4. GO enrichment analysis (Fig. 3c–e, respectively) showed the most significant downregulation of genes associated with positive regulation of I-kappaB kinase/NF-kappaB signalling (biological processes), as well as focal adhesion, regulation of cell shape, actin filament network, cytoskeleton (cellular components), and alpha-l-arabinofuranosidase activity (molecular function). Transcriptomic data (Supplementary Table 4) also confirmed differential expression of genes associated with the chr16p13.11 microduplication including *NDE1, NTAN1, PDXDC1*, and *ABCC1* that we had separately shown by RT-qPCR (Supplementary Figure 4b). We have also carried out differential expression analysis for each case separately compared to controls (Supplementary Tables 6–8). All cases exhibit a similar overall pattern of differential gene expression changes as discussed above for the pooled case data. Case 3, however, appears to also have a larger number of differentially expressed genes and a number of these appear to be related to the cell cycle.

RNA-seq data was also analysed to assess whether the set of differentially expressed genes we found in our cell studies was significantly enriched for miR-484 targets previously reported [25]. We used the miRWalk 2.0 database [40] (http://zmf.umm.uni-heidelberg.de/apps/zmf/mirwalk2/index.html) to obtain the 2588 gene targets of miR-484, which are predicted by at least 50% of the 12 prediction programmes used by the database. We then looked to see if our set of 1012 differentially expressed genes was enriched in miR-484 targets, as compared to the background set of genes expressed in our RNA-seq data. Fisher’s exact test (one-sided) gave a small enrichment, with an odds ratio (OR) of 1.38 (*p* = 0.003) for targets of miR-484 among the genes with adjusted *p*-value < 0.05. To determine whether enrichment of our key GO term (positive regulation of I-kappaB kinase/NF-kappaB signalling) and enrichment of miR-484 targets remains significant and maintained if we impose higher FPKM cut-offs we have plotted (i) the *p*-value for enrichment via GO analysis of the top downregulated GO term ‘Positive regulation of I-kappaB kinase/NF-kappaB signalling’ in differentially expressed genes above this cut-off versus all genes above this cut-off and (ii) the *p*-value for enrichment of miR-484 targets in differentially expressed genes above this cut-off versus all genes above this cut-off p-values plotted as −log10(p) (Supplementary Figure 6b). While the GO term ‘Positive regulation of I-kappaB kinase/NF-kappaB signalling’ enrichment (blue line) remains below *p* = 0.05 (as indicated by the black horizontal line, above which values are significant) as the FPKM cut-off increases, the miR-484 enrichment (red line) is already non-significant at a cut-off of FPKM > 0.5 (Supplementary Figure 6b).

**Fig. 4:**
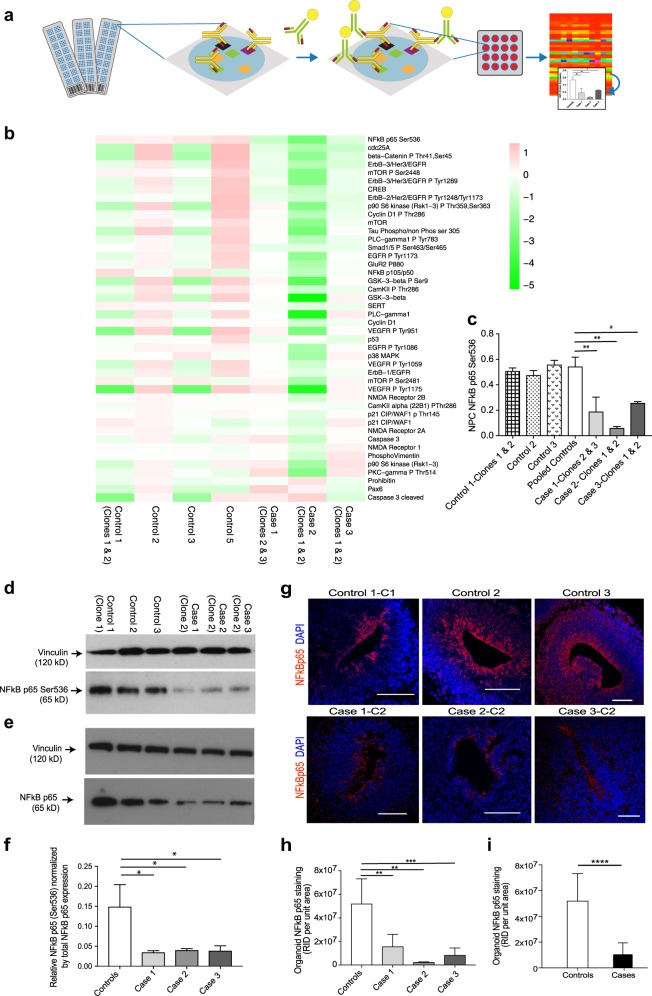
NF*κ*B p65 protein levels are significantly downregulated in NPCs and cerebral organoids from cases compared to controls. **a** Schematic overview of RPPA. **b** RPPA heatmap for the pooled data, i.e. each block is representing the value log2(protein abundance in line/mean abundance for this protein). Data are ordered by decreasing values of (mean protein abundance in cases)/(mean protein abundance in controls). Note that NF*κ*B appears as the top “downregulated” protein in the cases. **c** Graph of anti-phospho-NF*κ*B p65 (Ser536) expression in NPCs from case lines versus control lines determined by RPPA (Note that in the RPPA experiments we tested 2 clones for each of cases 1, 2 and 3; 2 clones for control 1; and one clone each from controls 2, 3 and 5. Each clone was run in triplicate). Statistics: mean  ± sem is shown; *p = 0.05; **p < 0.005 (ordinary one-way ANOVA with Dunnett’s multiple comparisons test). **d** Immunoblot of cell lysates from case-derived NPCs compared to controls stained with anti-phospho-NF*κ*B p65 (Ser536) and anti-vinculin loading control antibody. **e** Immunoblot of cell lysates from case-derived NPCs compared to controls stained with total NF*κ*B p65 antibody and anti-vinculin loading control antibody. **f** Quantification of immunoblots showing anti-phospho-NF*κ*B p65 (Ser536) normalized by total NF*κ*B p65 in pooled cases compared to controls. Statistics: mean  ± sem is shown; *p = 0.05 (ordinary one-way ANOVA with Dunnett’s multiple comparisons test). **g** Representative images of proliferative zones of 1-month-old organoids from individual control iPSC lines compared to individual case lines stained with anti-NF*κ*B p65 (red). Scale bar: 100 µm. **h** Graph of NF*κ*B p65 staining of cerebral organoids from three individual cases compared to three individual controls and also **i** graph of NF*κ*B p65 staining of cerebral organoids from pooled cases compared to pooled controls. Raw integrated density (the sum of the values of the pixels per unit area of organoid) was determined by capturing organoid images at the same magnification and calculated using Image J. Statistics: mean  ± sem is shown; **p < 0.005; ***p < 0.0005; ****p < 0.0001 (Mann–Whitney test)

Using reverse-phase proteomic array (RPPA) [32], we validated findings from the RNA-seq analysis in NPC lysates. Candidate antibody arrays were selected as per relevance to several of the aforementioned signalling pathways, in particular NF*κ*B and extracellular matrix signalling (Supplementary Table 4). Three replicates were tested in RPPA (Fig. 4a) for each cell line, plotted individually by antibody and displayed in a heat plot (Fig. 4b). Anti-phospho-NF*κ*B p65 (Ser536) showed the greatest differential expression of all proteins assayed, with pooled data revealing over 50% reduction in cases (Fig. 4c), confirmed also by immunoblotting with the anti-phospho-NF*κ*B p65 (Ser536) antibody (Fig. 4d). We also probed blots with an antibody to total NF*κ*B p65 (Fig. 4e) and show the relative phosphorylation levels at Ser536 normalized by total NF*κ*B p65 expression levels for each case compared to controls (Fig. 4f). All three cases show over 50% reduction compared to controls by quantification of immunoblots (Fig. 4f). Immunostaining showed that NF*κ*B p65 expression [as detected by two antibodies to total NF*κ*B p65 and an antibody specific to phospho-NF*κ*B p65 (ser536)] is localised to the apical surface of the ventricular zone in organoids (Fig. 4g and Supplementary Figure 5g-i), an area that co-localises with NPC proliferation and NDE1 expression (Supplementary Figure 5h-i). All organoids derived from cases had significantly less NF*κ*B p65 in the proliferative zones compared to organoids derived from controls (Fig. 4h, i; case organoids showed approximately 79.7% reduction in NF*κ*B p65 staining compared to control organoids; *p* < 0.0001, Mann–Whitney test).

### NPC proliferation deficits can be rescued by up-regulating expression of NFκB p65

**Fig. 5:**
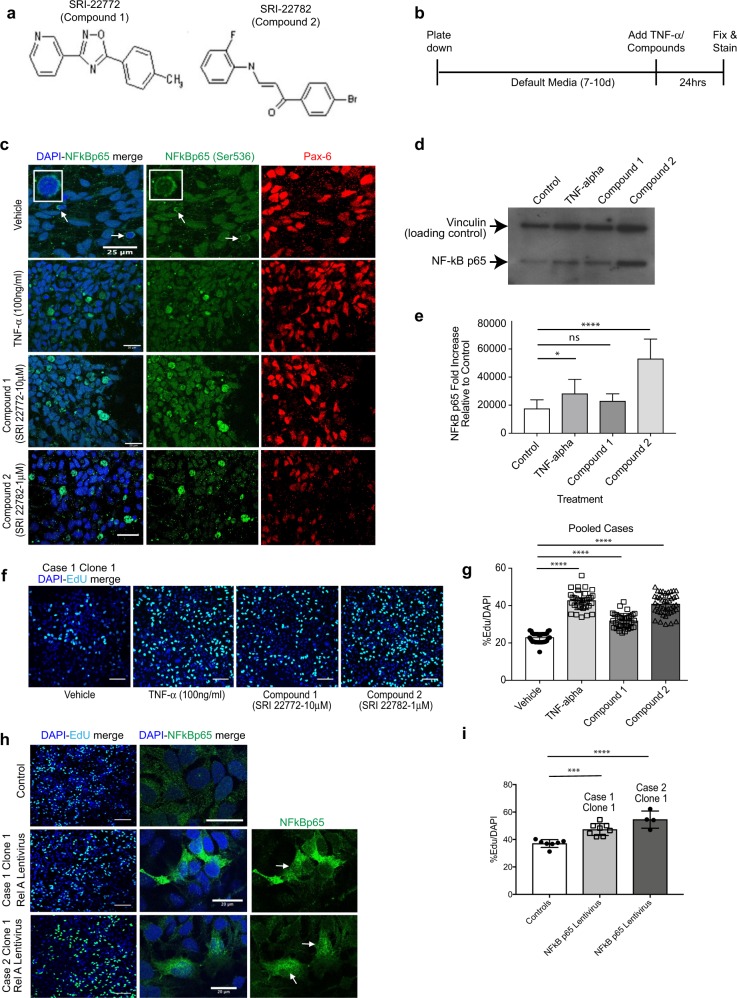
NF*κ*B p65 activator compounds cause p65 nuclear translocation in NPCs and reverse the proliferation deficit in case-derived NPCs. **a** Structures of NF*κ*B p65 activators compound 1 (SRI-22772) and compound 2 (SRI-22782). **b** NPCs (from controls and cases) were plated down in default media (without mitogens) and 7–10 days later TNF-α or compounds 1 or 2 were added to the media for 24 h. **c** NPCs treated with either TNF-α (positive control) or compounds 1 or 2 showed significantly increased anti-phospho-NF*κ*B p65 (Ser536) localising to the nucleus compared to vehicle-treated. **d** Nuclear extracts were prepared from NPCs treated with vehicle (DMSO control), TNF-α and compounds 1 and 2 and immunoblotted with anti-phospho-NF*κ*B p65 (Ser536). **e** Bands were quantified and normalised to the levels of vinculin (loading control). Compound 2 treatment resulted in significant increase in nuclear NF*κ*B p65 Ser536 expression (ordinary one-way ANOVA. Values plotted are the mean  ± sem; *p < 0.05; ns non-significant; ****p < 0.0001). **f** Representative images of proliferation assays of case NPCs (case 1, clone 1) following treatment with vehicle (DMSO), TNF-α, compounds 1 and 2. **g** Graph showing proliferation of all case NPCs (pooled) showing significant rescue of proliferation deficits following treatment with TNF-α and compounds 1 and 2 (ordinary one-way ANOVA with Dunnett’s multiple comparison test. Values plotted are the mean  ± sem; ****p < 0.0001). Representative images of case NPCs infected with NF*κ*B p65 (RELA) Lentivirus or vector alone (control) demonstrate that cells infected with RELA express increased NF*κ*B p65 and exhibit increased proliferation **i** compared to NPCs from the same patient infected with the vector alone

To investigate whether it is possible to rescue the NPC proliferation deficits by targeting the NF*κ*B p65 pathway we utilised recently described small molecular weight compounds that up-regulate NF*κ*B p65 expression and activity in a non-canonical, cytokine receptor-independent manner [34]. Two commercially available lead compounds, SRI-22772 and SRI-22782, were chosen. Both have heterocyclic structures and are denoted compound 1 and 2 for the purposes of this study (Fig. 5a). First, we treated case and control NPCs with both compounds at concentrations (10 and 1 μM, respectively) that had been shown to cause optimal non-canonical long-lasting NF*κ*B p65 activation [34]. We studied the ability of these two lead compounds to cause translocation of NF*κ*B p65 to the cell nucleus, using TNF-α as a positive control [34], in 2D NPC cultures (Fig. 5c). Following 24-h exposure to both compounds, there was significant NF*κ*B p65 nuclear translocation as detected by immunofluorescence that was confirmed by quantitative immunoblots of nuclear fractions (Fig. 5d, e). Moreover, compound 2 was significantly more effective at increasing nuclear NF*κ*B p65 compared to both TNF-α and compound 1. Having established functional efficacy of compounds 1 and 2 to activate NF*κ*B p65 signalling, we next asked whether treatment of 16p13.11 microduplication carrier-derived NPCs (from cases 1, 2 and 3) with compound 1 or 2 would correct the observed mutation-dependent reduction in NPC proliferation. EdU proliferation assays were undertaken following treatment of case NPCs with TNF-α alone, compounds 1 and 2 for 24 h (Fig. 5f, g shows pooled data; Supplementary Figure 7a-b shows representative images and graphs from each of the three individual cases studied). Compared to controls (DMSO vehicle-treated only), TNF-α and compounds 1 or 2 resulted in significantly increased NPC proliferation, back to a level that was observed in NPCs derived from healthy individuals (~35–40% EdU/DAPI over an 8-h time course of proliferation; *p* < 0.0001, ordinary one-way ANOVA with Dunnett’s multiple comparison test). We ruled out an alternate mode of action of these compounds by ectopically expressing NF*κ*B p65 in 16p13.11 microduplication carrier patient-derived NPCs using a lentivirus construct. Overexpression of NF*κ*B p65 produced an effect similar to that of compound 2 as judged by EdU uptake assay thus showing that a rescue of NF*κ*B p65 was sufficient to reverse the 16p13.11 microduplication dependent change in NPC proliferation (Fig. 5h, i).

## Discussion

Human iPSC models when applied to genetically stratified cohorts are powerful tools to dissect the molecular and cellular underpinnings of neurodevelopmental disorders [20, 41–44]. Here, we have undertaken linked imaging and stem cell studies in a local cohort with a rare highly penetrant 16p13.11 CNV that shows a striking threefold overrepresentation in SCZ cases compared to controls [3, 7]. This study demonstrates a potential role for genes within the chromosome 16p13.11 microduplication (and *NDE1* in particular) in human corticogenesis and implicates dysregulation of NF*κ*B as an important mechanistic pathway in neurodevelopmental disorders.

Until recently, the study of human brain development was severely restricted by availability of human post-mortem foetal tissue leading to a reliance on rodent models for mechanistic understanding of the role of NDE1 in regulating NPC proliferation, migration and cortical thickness. *Nde1* mutant mouse studies have revealed various phenotypes including thin superficial cortical layers (II to IV) [19] and catastrophic DNA double strand breaks concurrent with DNA replication, leading to p53-dependent apoptosis and reduced neurons in cortical layer II/III [45]. More recently knockdown in rat using in utero electroporation confirmed these findings and show that *Nde1* effects are pronounced on premitotic nuclear migration with specific effects on radial glial progenitor cells and on primary cilia [46]. It has recently been shown that in utero electroporation of NDE1 short hairpin RNA (shRNA) in embryonic rat brains causes apical interkinetic nuclear migration defects and that the severity of NDE1-associated microcephaly results not from defects in mitosis but rather the inability of neural progenitors to ever reach this stage [46]. This is consistent with our findings of reduced cortical thickness upon structural MRI, and decreased and abnormal (symmetric versus asymmetric) divisions of neural progenitors in human case-derived organoids. Our study emphasizes the importance of human experimental system to investigate neurodevelopmental disorders furthering what has previously been reported in animal studies by allowing us to directly link human MRI brain scanning data with human cellular and molecular data in both 2D and 3D cellular systems from the same patients. Specific findings in this study, that have not been reported in animal studies, is the identification of a potential role for NF*κ*B p65 in this process. Unbiased transcriptomic and proteomic data has shown significant differences in expression of NF*κ*B p65 between cases and controls and furthermore it has been possible to rescue this in vitro in human-derived cells both genetically and pharmacologically.

Several independent studies have implicated chromosome 16p13.11 microduplication and the *NDE1* gene as underlying contributors to mental illness and abnormal corticogenesis in humans [3, 21, 22] and rodent [9–14] models, respectively. More recent studies have suggested that a microRNA-484, encoded within the 5’UTR of *NDE1* may be the causative factor in the locus [8, 25], whereas others had previously proposed that *NDE1* and *NTAN1* were the two most likely genes to be responsible [3]. Our data overexpressing the *NDE1* gene alone (without the miR-484) in control human-derived NPCs shows that it is possible to recapitulate the proliferation deficit seen in case-derived lines suggesting that an extra copy of the *NDE1* gene is in itself responsible. Using our RNA-sequencing data, we also looked to see if our set of 1012 differentially expressed genes was enriched in miR-484 targets that have been previously described [25]. Fisher's exact test (one-sided) gave a small enrichment, with an odds ratio (OR) of 1.38 (*p* = 0.003) for targets of miR-484 among the genes with adjusted *p*-value < 0.05 in our RNA-sequencing data. However, it is possible that this result is confounded by other factors unrelated to the 16p13.11 microduplication. Differential gene expression with RNA-seq data has, in general, a greater likelihood to detect statistical significance for genes that are more highly expressed or which have longer transcripts; and it might be (to give one possible example) that genes highly expressed in the brain are more likely to be targets of miR-484. Indeed, if we impose a cut-off of mean abundance over all samples > 1 FPKM, then the enrichment for targets of miR-484 among the differentially expressed genes was no longer observed (OR = 0.77, *p* = 0.9797). However, one might also argue that, for example, the enrichment we see for the GO term ‘Positive regulation of I-kappaB kinase/NF-kappaB signalling’ in our differentially expressed genes might actually be because genes with that annotation also tend to be genes highly expressed in the brain. If this were the case, we would reasonably expect to see that this GO enrichment is maintained when we impose higher FPKM cut-offs. We have indeed demonstrated this where, for various mean FPKM cut-offs, we have plotted the *p*-value for enrichment via GO analysis of the top downregulated GO term ‘Positive regulation of I-kappaB kinase/NF-kappaB signalling’ in differentially expressed genes above this cut-off versus all genes above this cut-off and the *p*-value for enrichment of miR-484 targets in differentially expressed genes above this cut-off versus all genes above this cut-off [*p*-values are plotted as −log10(p)]. Enrichment of the GO term ‘Positive regulation of I-kappaB kinase/NF-kappaB signalling’ remains significant regardless of the cut-off, whereas the mir-484 enrichment is already non-significant at a cut-off of FPKM > 0.5. Taken together with our data showing that overexpression of NDE1 affects cell proliferation of NPCs, these data argue against the miR-484 being the causative factor for differentially expressed genes in human NPCs.

It is also possible that genes within the chromosome 16p13.11 locus exert their effect in combination with other genes elsewhere in the genome as has recently been proposed in 22q11.2 deletion syndrome where it has been suggested that additional rare CNVs, overlapping genes outside the 22q11.2 deletion region, contribute to schizophrenia risk supporting a multigenic hypothesis for schizophrenia [47]. In this regard, it is noteworthy that case 3 carries both the 16p13.11 microduplication and also a de novo loss-of-function mutation in *TSC2*, yet demonstrates a similar cellular phenotype to cases 1 and 2. Several published clinical case reports describe patients with a combination of two rare genetic variants, one of which includes 16p13.11 duplications [48, 49] that has led to the proposal of a ‘two-hit model’ in which the 16p13.11 duplication may contribute to the phenotype, not only as a single event but also in association with another mutation [48].

In this regard, it is also interesting to note that in terms of clinical phenotype case 3 is perhaps the most severely affected suffering from intellectual disability, ASD and teenage onset schizophrenia. All three diagnoses could be associated with either the 16p13.11 microduplication and / or the *TSC2* LoF mutation. Our RPPA data showed a reduction in expression of mTOR protein and also mTOR phosphorylated Serine 2448, in case 3 consistent with an interplay between AKT, TSC2 and the mTOR complex [50] and suggests the involvement of additional pathways in this case. One limitation of stem cell studies, involving rare highly penetrant variants associated with neurodevelopmental disorders, relates to the small numbers of affected carriers actually studied. Future collaborative studies comparing cellular phenotypes and neuroimaging from larger cohorts will be essential to confirm and extend the reported findings.

RNA-sequencing data showed the most significantly affected GO category in cases compared to controls was downregulation of genes associated with positive regulation of I-kappaB kinase/NF-kappaB signalling which has been implicated in neurogenesis [51–55]. Our RPPA, immunoblotting and immunostaining data with anti-NF*κ*B p65 antibodies confirmed these findings in human 2D NPC cultures and also in human 3D cerebral organoid cultures. The NF*κ*B family contains 5 members including RelA(p65), RelB, c-Rel, p50/p105 (NF*κ*B1) and p52/p100 (NF*κ*B2), which form various combinations of homodimers or heterodimers [56] activated through a series of signaling cascades. The most common form of NF*κ*B in the brain is a dimer of p50/p65 (RelA). Neurogenesis involves highly controlled proliferation, survival, migration and lineage differentiation of neural stem/progenitor cells (NSC/NPCs) and is tightly regulated by intrinsic and extrinsic factors. Embryonic p65^−/−^ knockout mouse-derived NPCs display significantly reduced growth compared to wild-type littermates, an effect attributed to a reduction in the number of proliferating cells [57]. NF*κ*B signaling also regulates the early differentiation of NSCs [58], with addition of TNFα to activate NF*κ*B signaling under proliferation conditions sufficient to induce neural differentiation of NSCs/NPCs [58]. The role of NF*κ*B in both proliferation and initial differentiation of NSCs highlights a novel potential target for neurodevelopmental disorders characterized by dysregulated NPC proliferation, neuronal specification and lamination. Under physiologic conditions, moderate activation of NF*κ*B signaling promotes NSC differentiation into NPCs and maintains a continuous source for neurogenesis. Transgenic mouse models have also highlighted that NF*κ*B in NSCs/NPCs is necessary for axogenesis and maturation [59] yet, persistent and repeated overactivation of NF*κ*B signaling in NSCs may exhaust the NSC pool and thus lead to reduced neurogenesis as seen in aging [60] and chronic stress [61]. NF*κ*B is not only important in neurogenesis but a growing literature shows its importance in glial biology and myelination in both the CNS and PNS [52–55].

Interestingly, maternal immune activation models such as the polyIC mouse model also show activation of NF*κ*B following a single injection of polyIC during early gestational stages leading to dysregulated neural progenitor proliferation amongst other effects [62]. In patients with schizophrenia the expression of NF*κ*B-responsive genes is increased [63], and identification of three single-nucleotide polymorphism variants of the p65 gene associated with schizophrenia in a Japanese population provides further indirect evidence implicating this pathway in neurodevelopmental disorders [63]. Schizophrenia genome-wide association studies (GWAS) have consistently implicated genetic variants in the major histocompatibility complex (MHC) on chromosome 6 as a susceptibility factor suggesting that the immune abnormalities in schizophrenia may have a genetic origin [64]. It has long been suggested that stress, trauma and infection from conception onwards are important aetiological risk factors in the development of schizophrenia [65]. Chronic inflammation from the aforementioned causes can activate the immune system causing dysregulated NF*κ*B activation (the master switch) resulting in excessive and prolonged cytokine production, decreased neurogenesis, increased oxidative stress and apoptosis and glutamatergic activation bringing about structural abnormalities as well as alterations in brain rewiring. CNVs, such as those present in the chromosome 16p13.11 microduplication, for example increased *NDE1* expression, may increase risk by changing the expression of dosage-sensitive genes which themselves are either activators of or are regulated by an NF*κ*B pathway gone awry. The specific validation and mechanistic challenge of these genes within the 16p13.11 microduplication, both individually and in combination gene sets, is an imposing yet potentially rewarding investigation for the future to dissect the precise mechanism linking the NF*κ*B signaling pathway with neuropsychiatric and neurodevelopmental disorders and may drive the development of novel therapies [66].

## Electronic supplementary material

Supplementary Figure 5

Supplementary Figure 6

Supplementary Figure 7

Supplementary Table 2

Supplementary Table 3

Supplementary Table 4

Supplementary Table 1

Supplementary Figure 4

Supplementary Figure 2

Supplementary Figure 3

Supplemental Legends

Supplementary Figure 1

Supplementary Table 5

Supplementary Table 6

Supplementary Table 7

Supplementary Table 8

Supplementary Methods
