# Supplementary figures and images for: Reversal of proliferation deficits caused by chromosome 16p13.11 microduplication through targeting NFκB signaling: an integrated study of patient-derived neuronal precursor cells, cerebral organoids and in vivo brain imaging
Source: Mol Psychiatry. 2018 Nov 6;24(2):294–311. doi: 10.1038/s41380-018-0292-1 (PMC6344377; doi:10.1038/s41380-018-0292-1)

Supplementary Figure 5

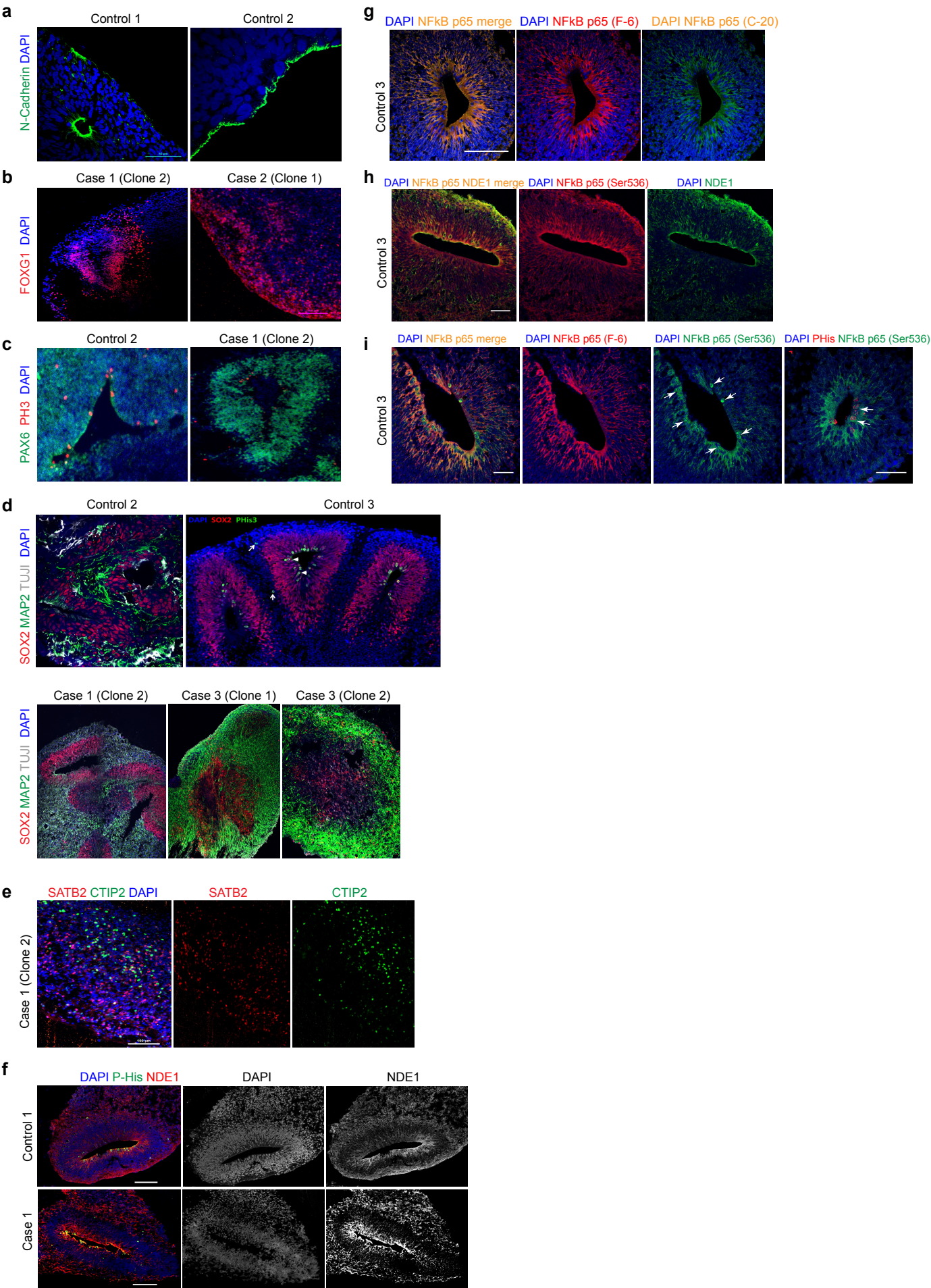

Supplement: Supplementary file 1 — Supplementary Figure 5 [file 41380_2018_292_MOESM1_ESM.pdf]

Supplementary Figure 6

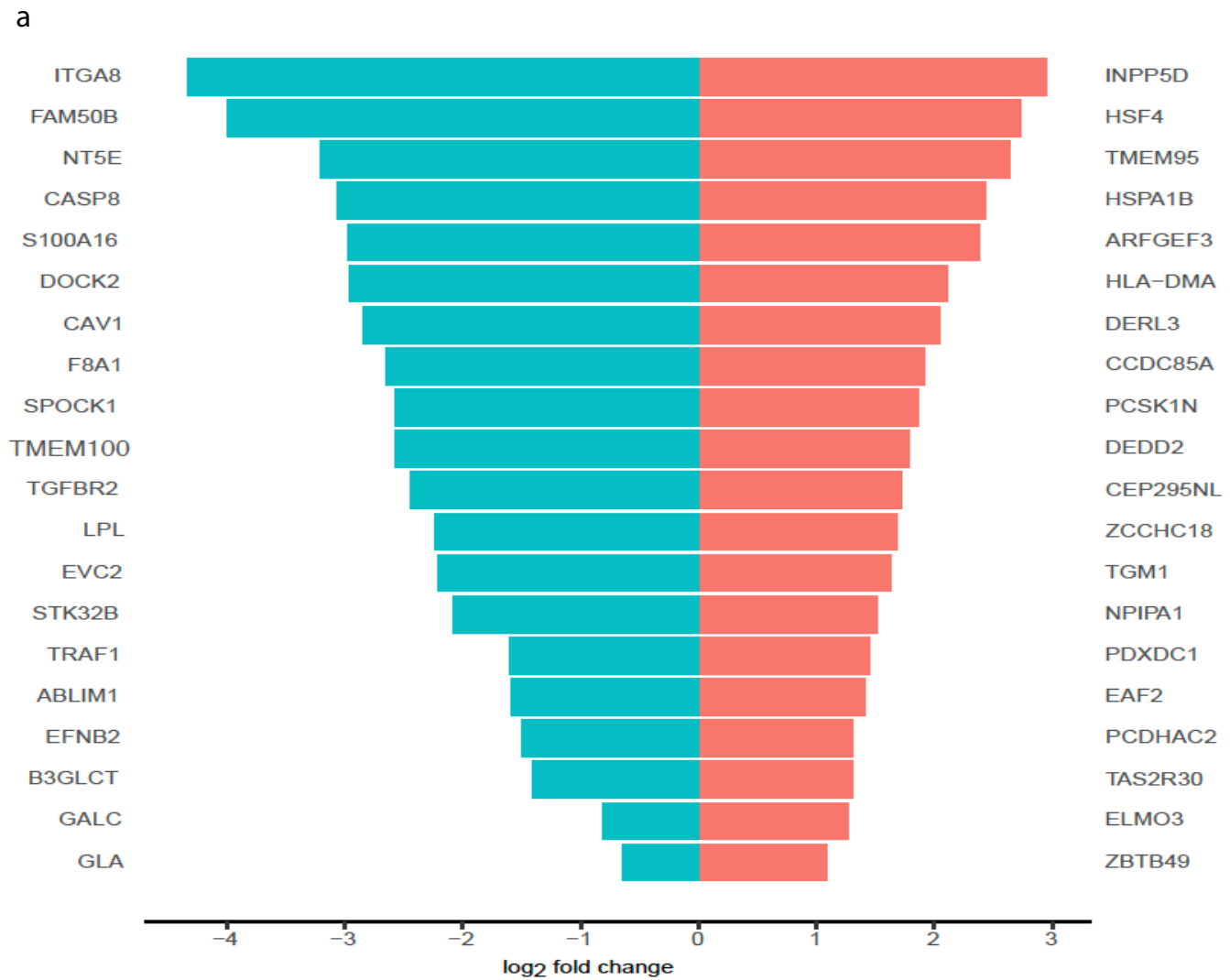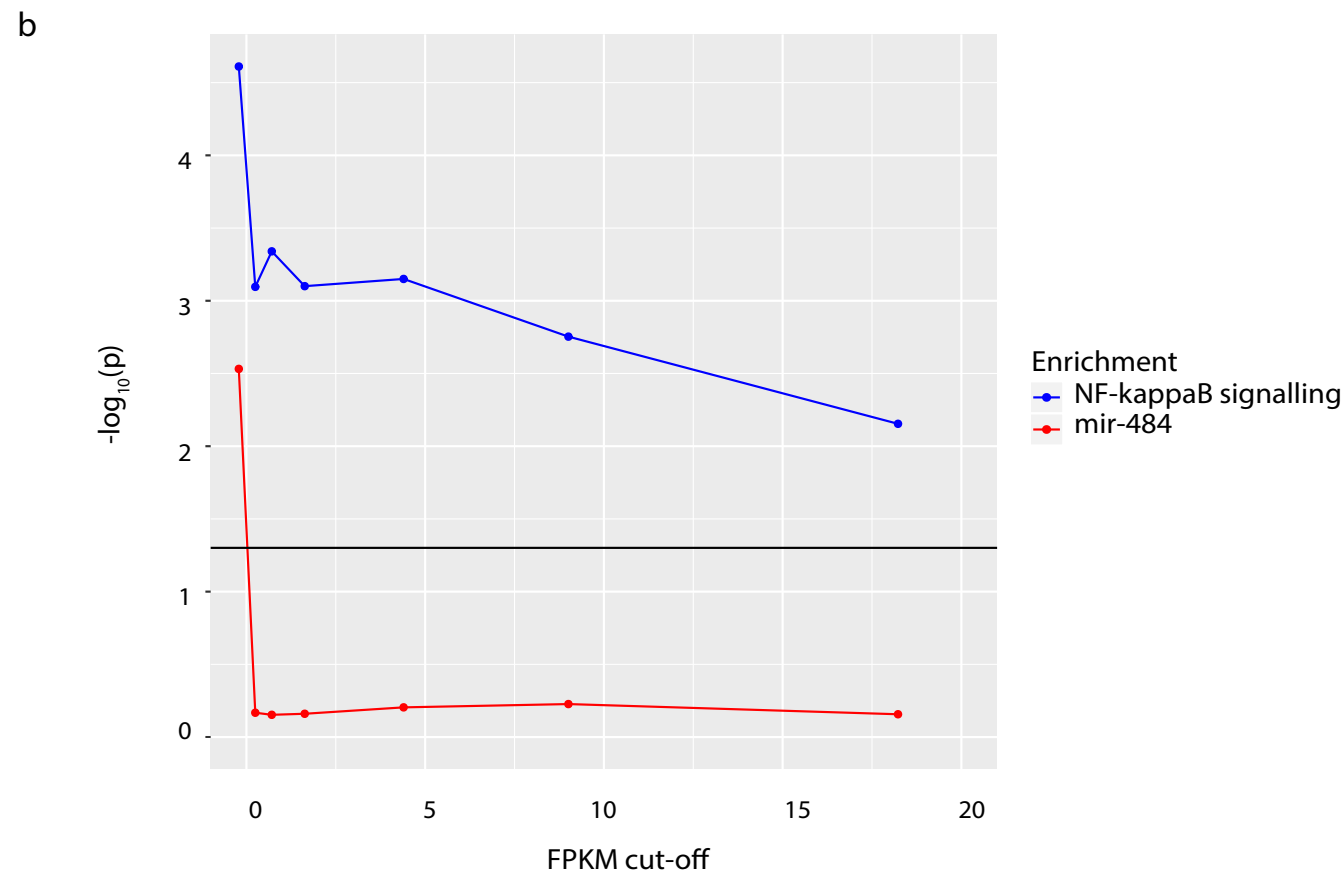

Supplement: Supplementary file 2 — Supplementary Figure 6 [file 41380_2018_292_MOESM2_ESM.pdf]

Supplementary Figure 7

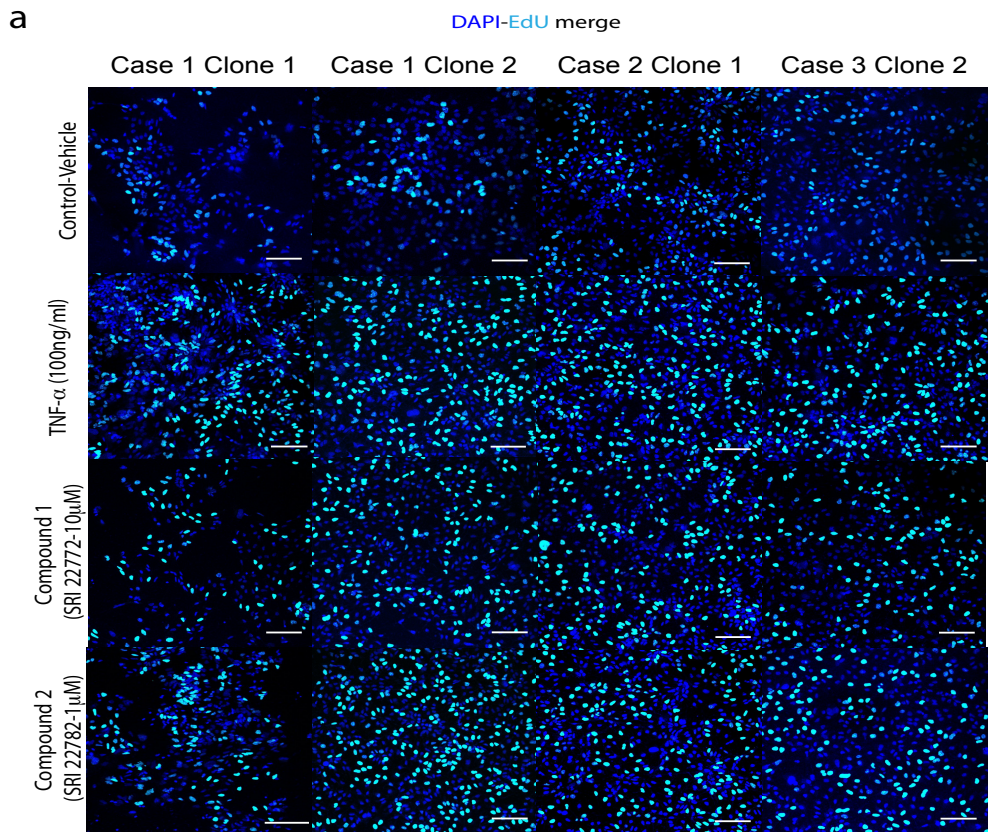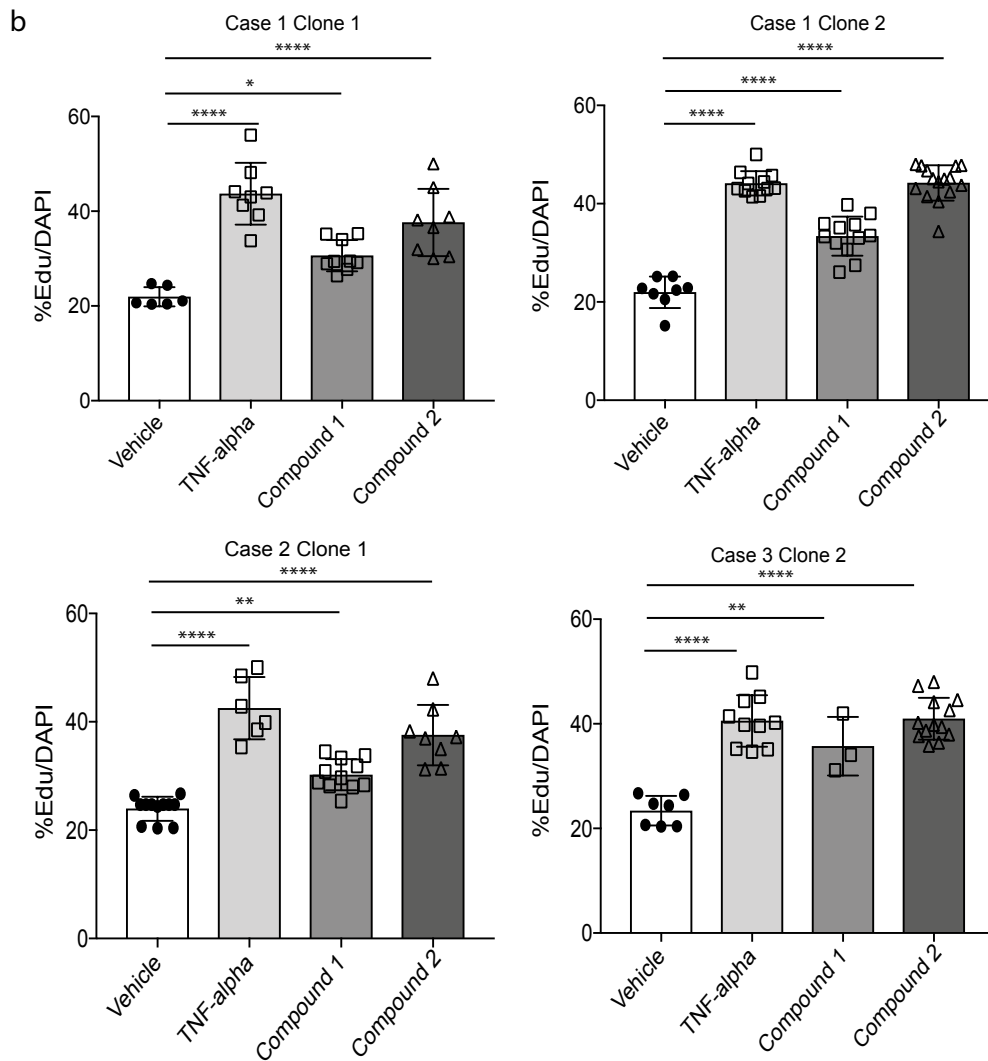

Supplement: Supplementary file 3 — Supplementary Figure 7 [file 41380_2018_292_MOESM3_ESM.pdf]

Supplementary Figure 4

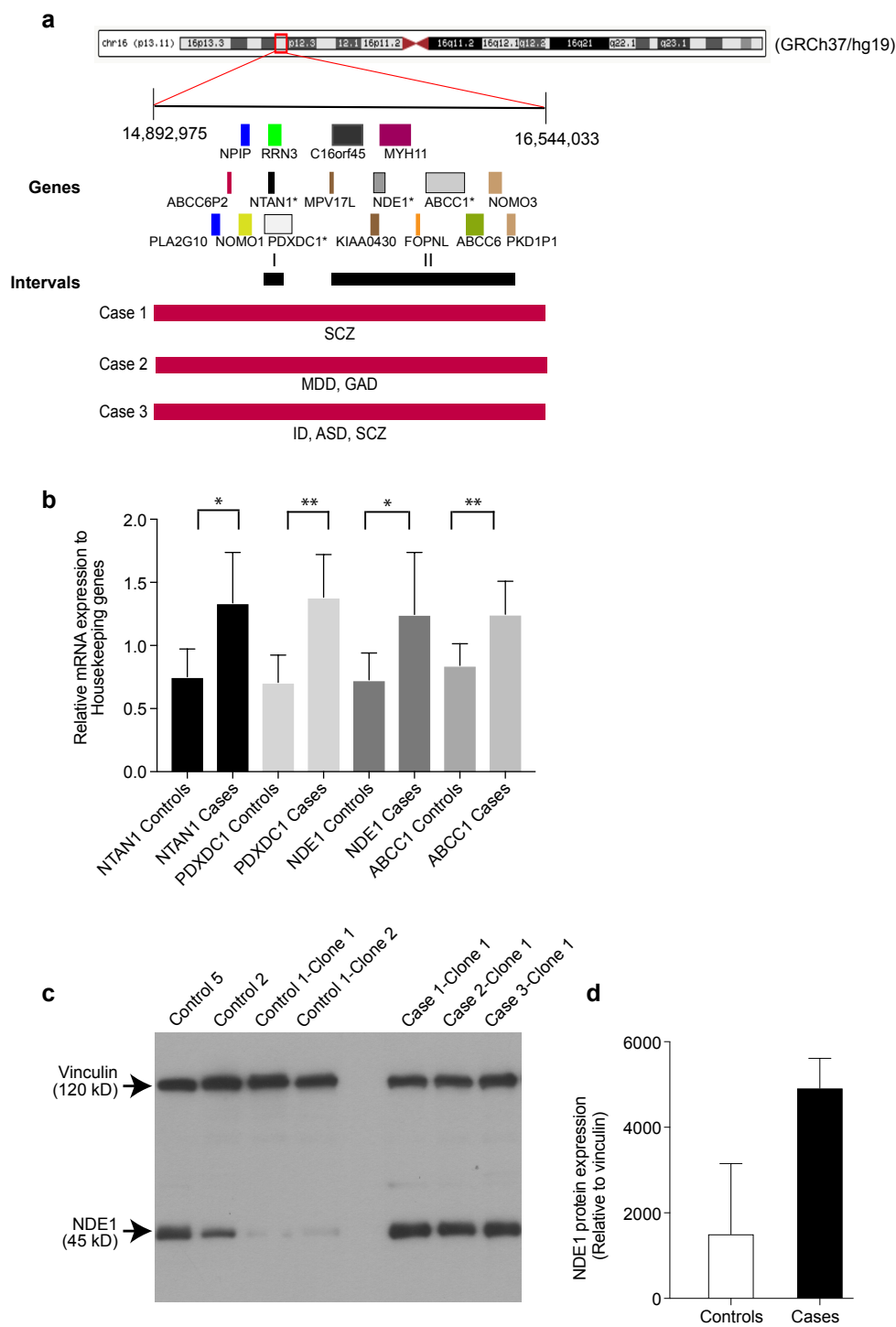

Supplement: Supplementary file 8 — Supplementary Figure 4 [file 41380_2018_292_MOESM8_ESM.pdf]

Supplementary Figure 2

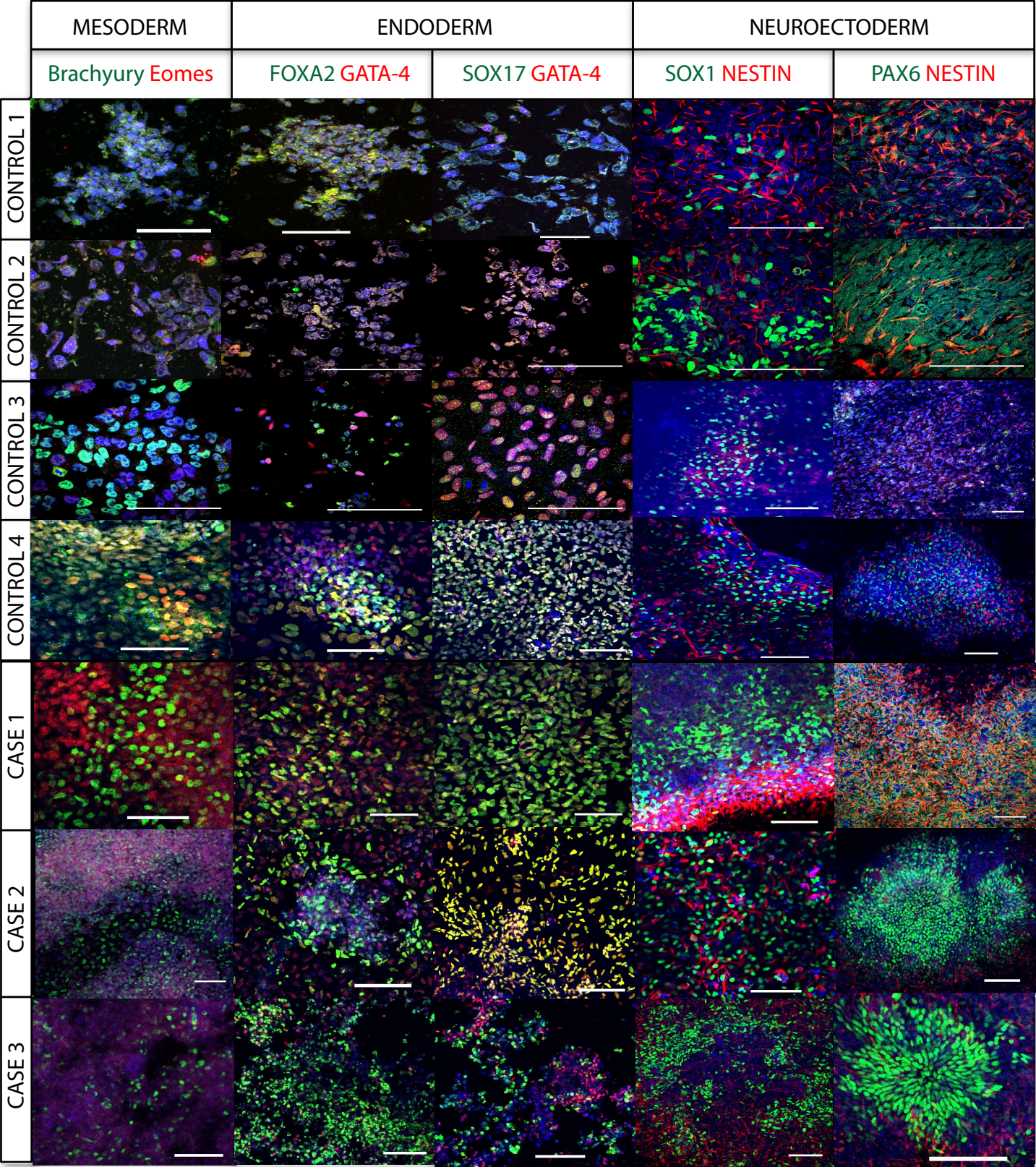

Supplement: Supplementary file 9 — Supplementary Figure 2 [file 41380_2018_292_MOESM9_ESM.pdf]

Supplementary Figure 3

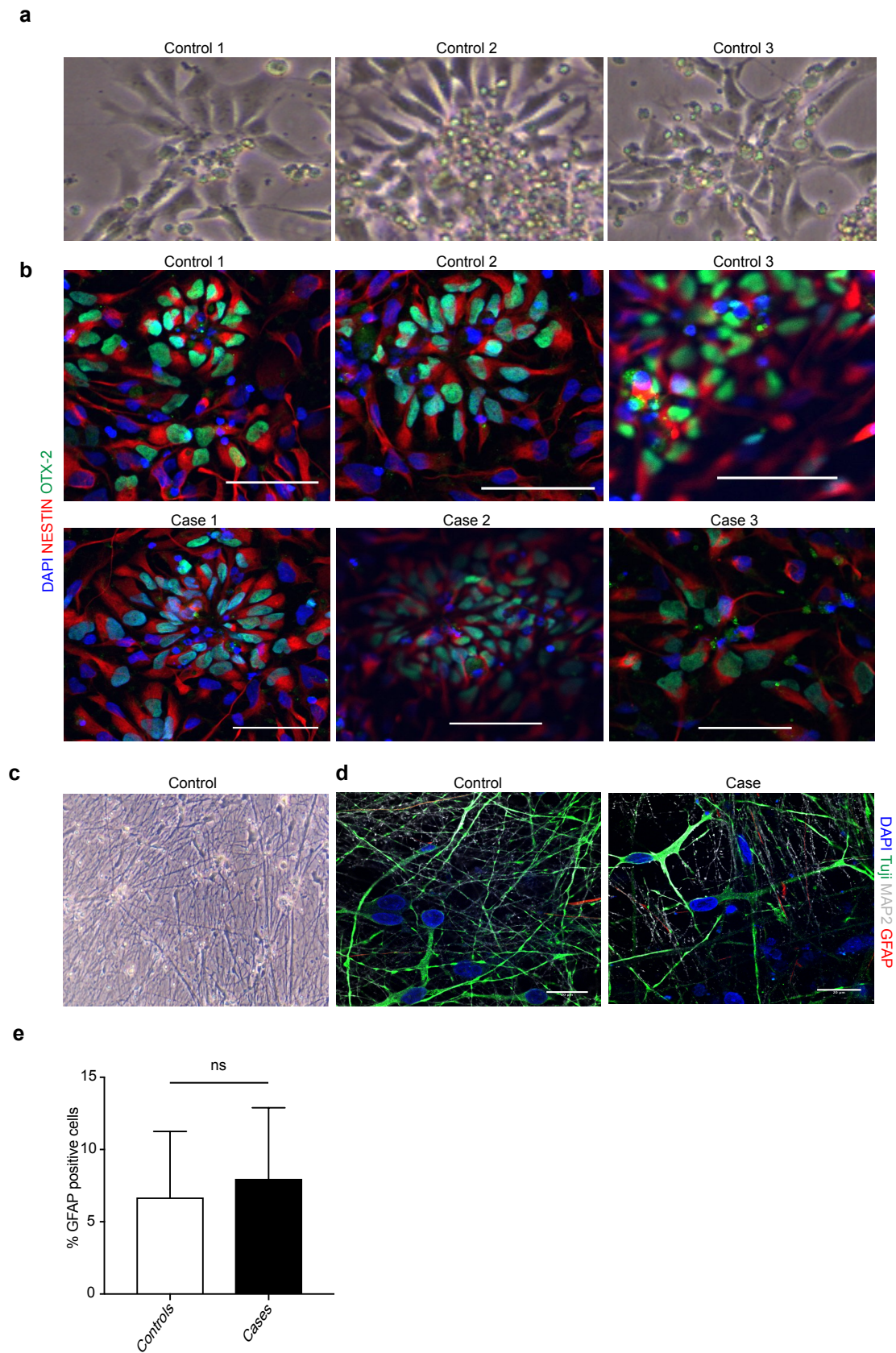

Supplement: Supplementary file 10 — Supplementary Figure 3 [file 41380_2018_292_MOESM10_ESM.pdf]

Supplementary Figure 1

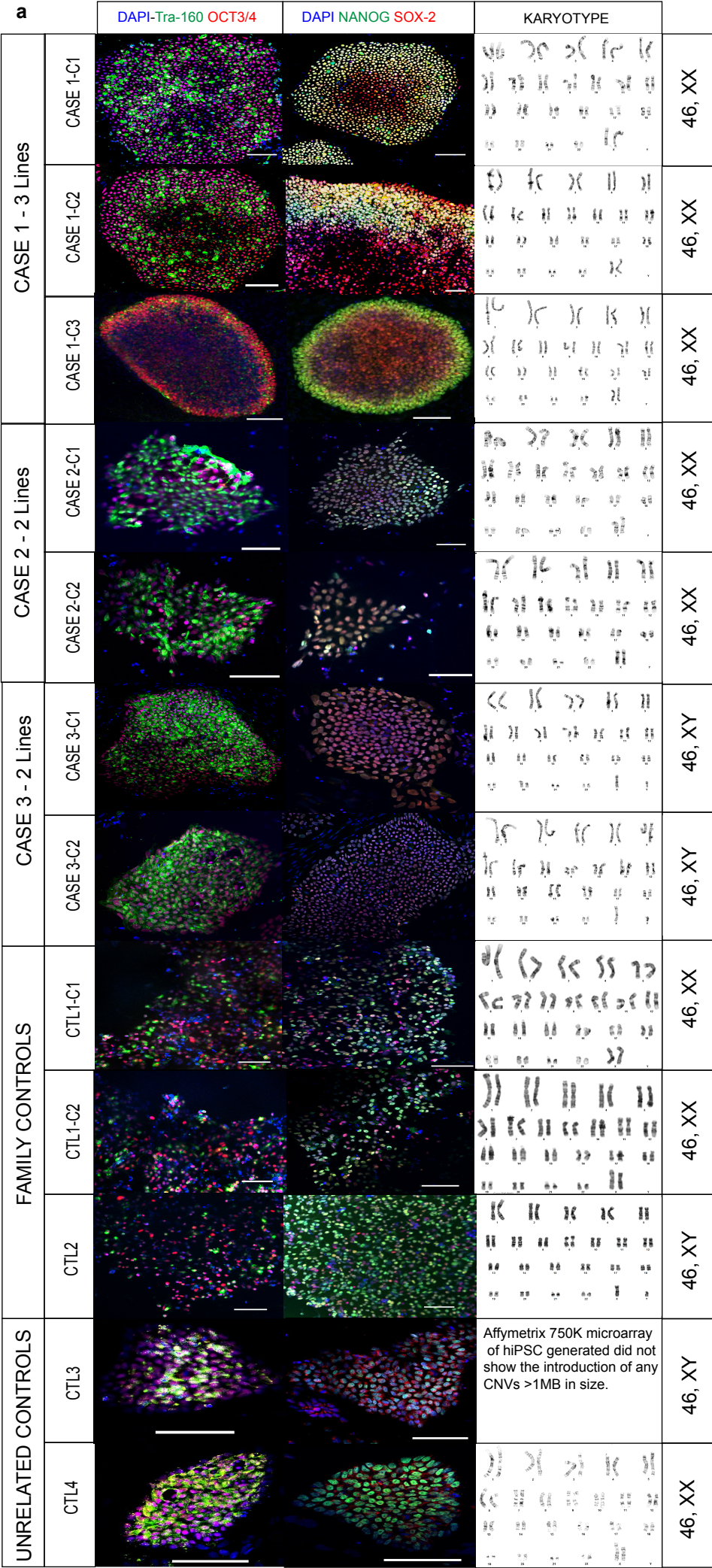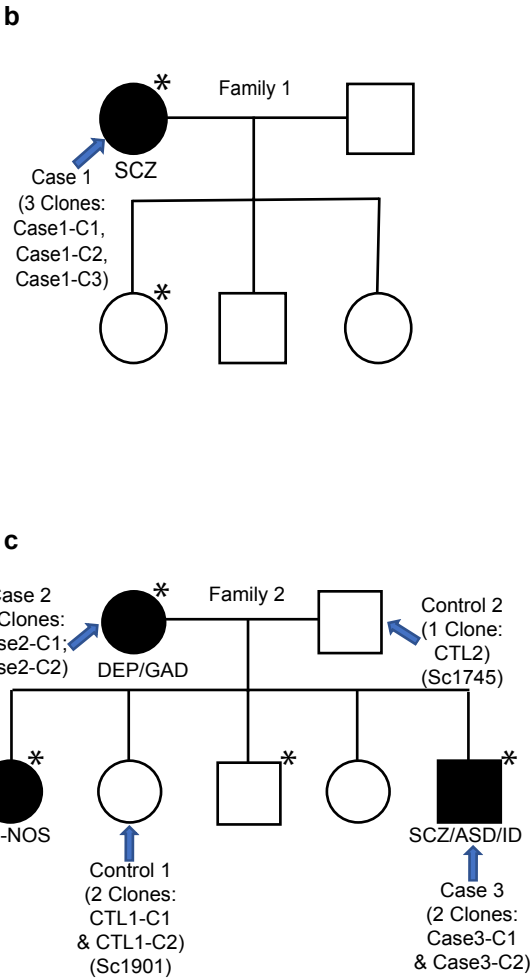

Supplement: Supplementary file 12 — Supplementary Figure 1 [file 41380_2018_292_MOESM12_ESM.pdf]
