# Supplementary material for: Reversal of proliferation deficits caused by chromosome 16p13.11 microduplication through targeting NFκB signaling: an integrated study of patient-derived neuronal precursor cells, cerebral organoids and in vivo brain imaging
Source: Mol Psychiatry. 2018 Nov 6;24(2):294–311. doi: 10.1038/s41380-018-0292-1 (PMC6344377; doi:10.1038/s41380-018-0292-1)
Supplement: Supplementary file 4 — Supplementary Table 2 [file 41380_2018_292_MOESM4_ESM.pdf]

Supplementary Table S2: List of antibodies used

Antibody	Host	Company	Cat. No.	Working Dilution
**β-ACTIN**	Mouse monoclonal IgG2b	ThermoFisher	MA5-15739	1:1,000 (WB)
**Brachyury**	Goat polyclonal	R&D Systems	AF2085	1:100 (ICC)
**CTIP2**	Rat monoclonal IgG2a	Abcam	ab18465	1:500 (ICC)
**FOXA2**	Goat polyclonal	R&D Systems	AF2400	1:100 (ICC)
**FOXG1**	Rabbit polyclonal	Abcam	ab18259	1:500 (ICC)
**GATA-4**	Mouse monoclonal IgG2a	Santa Cruz	SC-25310	1:100 (ICC)
**GFAP**	Rabbit polyclonal	Dako	Z0334	1:500 (ICC)
**Histone H3 (phospho S10)**	Mouse monoclonal IgG	Abcam	ab14955	1:300 (ICC)
**MAP2**	Mouse monoclonal IgG1	Sigma	M9942	1:500 (ICC)
**NANOG**	Goat monoclonal IgG1	R&D Systems	AF1997	1:100 (ICC)
**N-Cadherin**	Mouse monoclonal IgG1	BD Biosciences	610920	1:500 (ICC)
**NDE1**	Rabbit polyclonal	Proteintech	10233-1-AP	1:100 (ICC)<br>1:500 (WB)
**NESTIN**	Mouse monoclonal IgG1	Millipore	MAB5326	1:100 (ICC)
**Phosph-NF-κB p65 (Ser536)**	Rabbit monoclonal IgG1	Cell Signaling	3033	1:100 (ICC)<br>1:1,000 (WB)
**NF-κB p65 (C-20)**	Rabbit polyclonal	Santa Cruz	SC-372	1:100 (ICC)<br>1:300 (WB)
**NF-κB p65 (F-6)**	Mouse monoclonal	Santa Cruz	SC-8008	1:100 (ICC)<br>1:200 (WB)
**Oct3/4 (C-10)**	Mouse monoclonal IgG2B	Santa Cruz	SC-5279	1:250 (ICC)
**OTX-2**	Goat polyclonal	R&D Systems	AF1979	1:100 (ICC)
**Pax-6**	Rabbit polyclonal	BioLegend	901301	1:300 (ICC)
**Reelin (clone 142)**	Mouse monoclonal IgG1	Millipore	MAB5366	1:200 (ICC)
**SATB2**	Mouse monoclonal IgG1	Abcam	ab51502	1:100 (ICC)
**SOX1**	Goat polyclonal	R&D Systems	AF3369	1:100 (ICC)
**SOX2**	Rabbit polyclonal	Abcam	ab97959	1:100 (ICC)
**SOX17**	Goat polyclonal	R&D Systems	AF1924	1:200 (ICC)
**Tbr2**	Rabbit polyclonal	Millipore	AB2283	1:300 (ICC)
**Tra-1-60**	Mouse monoclonal IgM	Stemgent	09-0010	1:100 (ICC)
**β-III Tubulin**	Mouse monoclonal (isotype III)	Sigma	T5076	1:100 (ICC)
**Phosphorylated Vimentin (Ser55)**	Mouse monoclonal IgG2B	MBL	D076-3	1:300 (ICC)
**Vinculin**	Rabbit monoclonal	Abcam	ab129002	1:10,000 (WB)
