# Supplementary material for: Reversal of proliferation deficits caused by chromosome 16p13.11 microduplication through targeting NFκB signaling: an integrated study of patient-derived neuronal precursor cells, cerebral organoids and in vivo brain imaging
Source: Mol Psychiatry. 2018 Nov 6;24(2):294–311. doi: 10.1038/s41380-018-0292-1 (PMC6344377; doi:10.1038/s41380-018-0292-1)
Supplement: Supplementary file 5 — Supplementary Table 3 [file 41380_2018_292_MOESM5_ESM.pdf]

Cell Line	NPC proliferation studies (Fig 2a)	Organoid studies (Fig 2g-I & Fig 4g-i)	RNA sequencing studies	Proteomics (RPPA & WB)(Fig 4b-f)	NFκB activator screen (Fig 5f-g)	Lentivirus RELA expression (Fig 5h-i)
Case 1-C1	✓	-	-	-	✓	✓
Case 1-C2	✓	✓	✓	✓	✓	-
Case 1-C3	-	-	✓	✓	-	-
Case 2-C1	✓	-	-	✓	✓	✓
Case 2-C2	✓	✓	✓	✓	-	-
Case 3-C1	✓	✓	-	✓	-	-
Case 3-C2	✓	✓	✓	✓	✓	-
CTL1-C1	✓	✓	✓	✓	-	-
CTL1-C2	-	-	✓ (2 NPC derivations)	✓	-	-
CTL2	✓	✓	✓	✓	-	-
CTL3	✓	✓	✓ (2 NPC derivations)	✓	-	-
CTL4	-	-	✓	-	-	-
CTL5	✓	-	✓	✓	-	-

Supplementary Table S3: Cell lines used in the various experiments of this study
