# Supplementary material for: Reversal of proliferation deficits caused by chromosome 16p13.11 microduplication through targeting NFκB signaling: an integrated study of patient-derived neuronal precursor cells, cerebral organoids and in vivo brain imaging
Source: Mol Psychiatry. 2018 Nov 6;24(2):294–311. doi: 10.1038/s41380-018-0292-1 (PMC6344377; doi:10.1038/s41380-018-0292-1)
Supplement: Supplementary file 6 — Supplementary Table 4 [file 41380_2018_292_MOESM6_ESM.pdf]

Supplementary Table S4: Antibodies used in RPPA study

Antibody	Supplier	Catalog Number	Species
PARP	Cell Signaling Technologies	9542	rabbit
p44/42 MAPK (ERK1/2)	Cell Signaling Technologies	9102	rabbit
p44/42 MAPK (ERK1/2) P Thr202/Thr185,Tyr204	Cell Signaling Technologies	4370	rabbit
Akt	Cell Signaling Technologies	9272	rabbit
Akt P Ser473	Cell Signaling Technologies	4060	rabbit
PARP cleaved Asp214	Cell Signaling Technologies	9541	rabbit
beta-actin	Cell Signaling Technologies	4970	rabbit
beta-Catenin	Cell Signaling Technologies	9562	rabbit
beta-Catenin P Ser33,Ser37,Thr41	Cell Signaling Technologies	9561	rabbit
PTEN	Cell Signaling Technologies	9552	rabbit
PTEN P Ser380,Thr382,Thr383	Cell Signaling Technologies	9554	rabbit
SAPK/JNK	Cell Signaling Technologies	9258	rabbit
GSK-3-alpha/beta P Ser21/Ser9	Cell Signaling Technologies	9331	rabbit
p53 P Ser15	Cell Signaling Technologies	9284	rabbit
p38 MAPK PThr180,Tyr182	Cell Signaling Technologies	9211	rabbit
p38 MAPK	Cell Signaling Technologies	9212	rabbit
mTOR P Ser2448	Cell Signaling Technologies	2971	rabbit
mTOR	Cell Signaling Technologies	2972	rabbit
p90 S6 kinase (Rsk1-3) P Thr359,Ser363	Cell Signaling Technologies	9344	rabbit
Smad1/5 P Ser463/Ser465	Cell Signaling Technologies	9516	rabbit
Cyclin D1 P Thr286	Cell Signaling Technologies	3300	rabbit
Caspase 3	Cell Signaling Technologies	9662	rabbit
Caspase 3 cleaved	Cell Signaling Technologies	9664	rabbit
p53	Cell Signaling Technologies	9282	rabbit
GSK-3-beta P Ser9	Cell Signaling Technologies	9336	rabbit
GSK-3-beta	Cell Signaling Technologies	9315	rabbit
Prohibitin	Santa Cruz	sc-28259	rabbit
p21 CIP/WAF1 p Thr145	Santa Cruz	20220-R	rabbit
p90 S6 kinase (Rsk1-3)	Santa Cruz	sc-231	rabbit
p21 CIP/WAF1	Cell Signaling Technologies	2946	mouseIgG2a
CamKII P Thr286	Cell Signaling Technologies	3361	rabbit
ErbB-1/EGFR	Cell Signaling Technologies	2232	rabbit
ErbB-2/Her2/EGFR P Tyr1248/Tyr1173	Cell Signaling Technologies	2244	rabbit
ErbB-3/Her3/EGFR	Cell Signaling Technologies	4754	rabbit
ErbB-3/Her3/EGFR P Tyr1289	Cell Signaling Technologies	4791	rabbit
EGFR P Tyr1173	Cell Signaling Technologies	4407	rabbit
NFkB p65 Ser536	Cell Signaling Technologies	3033	rabbit
beta-Catenin P Thr41,Ser45	Cell Signaling Technologies	9565	rabbit
cdc25A	Cell Signaling Technologies	3652	rabbit
VEGFR P Tyr1175	Cell Signaling Technologies	2478	rabbit
Cyclin D1	Cell Signaling Technologies	2926	mouseIgG2a
PLC-gamma1 P Tyr783	Cell Signaling Technologies	2821	rabbit
CREB	Cell Signaling Technologies	9197	rabbit
EGFR P Tyr1086	Cell Signaling Technologies	369700	rabbit
PLC-gamma1	Cell Signaling Technologies	2822	rabbit
Tau	Cell Signaling Technologies	1178-1	rabbit
VEGFR P Tyr951	Cell Signaling Technologies	4991	rabbit
Tau Phospho/non Phos ser 305	Cell Signaling Technologies	2368-1	rabbit
VEGFR P Tyr1059	Cell Signaling Technologies	3817	rabbit
NFkB p105/p50	Calbiochem	GTX110585	rabbit
ErbB-2/Her2/EGFR	Merck (Calbiochem)	PC116	rabbit
CamKII alpha (22B1) P Thr286	Abcam	ab7260	mouseIgG1
GFAP	Abcam	ab6046	rabbit
beta-Tubulin	Abcam	ab32502	rabbit
PKC-gamma P Thr514	Millipore (Upstate)	09-343SP	rabbit
mTOR P Ser2481	Cell Signaling Technologies	2405	rabbit
