# Supplementary material for: Reversal of proliferation deficits caused by chromosome 16p13.11 microduplication through targeting NFκB signaling: an integrated study of patient-derived neuronal precursor cells, cerebral organoids and in vivo brain imaging
Source: Mol Psychiatry. 2018 Nov 6;24(2):294–311. doi: 10.1038/s41380-018-0292-1 (PMC6344377; doi:10.1038/s41380-018-0292-1)
Supplement: Supplementary file 7 — Supplementary Table 1 [file 41380_2018_292_MOESM7_ESM.pdf]

Supplementary Table S1

Subject	Sex	Diagnosis	Genotype	Psychotropic Medications (Prescribed at time of biopsy)	Age at Onset/ Age at Biopsy/ Age at MRI
Control 1	Female (Daughter of Case 2 & Sister of case 3)	Unaffected	Non-Carrier	None	NA/ 29 yo/ 30 yo
Control 2	Male (Spouse of case 2 & Father of case 3)	Unaffected	Non-Carrier	None	NA/ 55 yo/ 55 yo
Control 3	Male	Unaffected control from Scottish t1:11 *DISC1* family	Non-Carrier (ID No. 40 from Suppl. Ref. 4)	None	NA/ 75 yo/ 75 yo
Control 4	Female	Unaffected control from Scottish t1:11 *DISC1* family	Non-Carrier (ID No. 28 from Suppl. Ref. 4)	None	NA/ 52 yo/ 52 yo
Control 5	Female	Unaffected	Non-Carrier	None	NA/ 46 yo/ NA
Case 1	Female	Paranoid schizophrenia	16p13.11 dup carrier	Olanzapine	14 yo (psychosis)/ 47 yo/ 47 yo
Case 2	Female (Affected mother of case 3)	Generalised anxiety disorder/ recurrent depression	16p13.11 dup carrier	Amitriptyline	18 yo (Depression)/ 48 yo/ 50 yo
Case 3 [UK10K_MUIR1523898]	Male (Son of case 2 and control 2)	Paranoid schizophrenia, intellectual disability, tuberous sclerosis, autism	16p13.11 dup carrier/*TSC2* LOF mutation (UK10K sequencing study: found to have a LoF variant in *TSC2* at 16:2115634:C/T. This is a stop-gained de novo variant in the *tuberin* gene, linked to tuberous sclerosis)	Olanzapine Sodium Valproate Fluoxetine Diazepam	18 yo (psychosis) 24 yo/ 26 yo

Supplementary Table S1

Extended clinical details:
Case 1: Diagnosed with paranoid schizophrenia at age 14 years. Experiences auditory hallucinations, paranoia and thought disorder.
Case 2: Diagnosed with depression in her late teens and has suffered multiple recurrent episodes over the years. Also suffers from generalised anxiety disorder.
Case 3: Experiences command hallucinations, low mood, reduced motivation and poor self-care. First diagnosed with depression at 18 years of age and found to be psychotic. Responding to unseen stimuli, paranoid, agitated and aggressive when unwell. Four episodes of psychosis in past 5 years with multiple inpatient psychiatric admissions. Head CT's have not shown any associated tubers although known to have a *de novo* loss-of-function mutation in *TSC2*. EEGs within normal limits.
