# Supplementary material for: Reversal of proliferation deficits caused by chromosome 16p13.11 microduplication through targeting NFκB signaling: an integrated study of patient-derived neuronal precursor cells, cerebral organoids and in vivo brain imaging
Source: Mol Psychiatry. 2018 Nov 6;24(2):294–311. doi: 10.1038/s41380-018-0292-1 (PMC6344377; doi:10.1038/s41380-018-0292-1)
Supplement: Supplementary file 11 — Supplemental Legends [file 41380_2018_292_MOESM11_ESM.docx]

## SUPPLEMENTARY FIGURES

**Supplementary Table S1.**

Table of clinical characteristics of participants included in this study.

## Supplementary Table S2.

Antibodies used in this study including suppliers and working dilutions.

## Supplementary Table S3.

Overview of the cell lines used in the respective experiments in this study.

## Supplementary Table S4.

Antibodies used in RPPA studies and suppliers.

## Supplementary Table S5.

RNA sequencing data of NPCs from 16p13.11 microduplication from all 3 cases compared to controls. Excel spreadsheet containing only those genes that were called significant at FDR < 0.05, with columns listing Ensembl ID, gene name, description, mean FPKM in each of the two conditions, mean FPKM across all 12 samples, the DESeq2 adjusted p-value, and a fold change column. The top 20 downregulated (in bold green text) and top 20 up- regulated (in bold orange text) genes are highlighted.

## Supplementary Table S6.

RNA sequencing data of NPCs from 16p13.11 microduplication case 1 compared to controls. Excel spreadsheet containing only those genes that were called significant at FDR

< 0.05, with columns listing Ensembl ID, gene name, description, mean FPKM in each of the two conditions, the DESeq2 adjusted p-value, and a fold change column.

## Supplementary Table S7.

RNA sequencing data of NPCs from 16p13.11 microduplication case 2 compared to controls. Excel spreadsheet containing only those genes that were called significant at FDR

< 0.05, with columns listing Ensembl ID, gene name, description, mean FPKM in each of the two conditions, the DESeq2 adjusted p-value, and a fold change column.

## Supplementary Table S8.

RNA sequencing data of NPCs from 16p13.11 microduplication case 3 compared to controls. Excel spreadsheet containing only those genes that were called significant at FDR

< 0.05, with columns listing Ensembl ID, gene name, description, mean FPKM in each of the two conditions, the DESeq2 adjusted p-value, and a fold change column.

## Supplementary Figure 1.

Characterization of iPSC lines with or without 16q13.11 dup. (a) Sample images of immunostaining of different iPSC lines. Note that all iPSC lines were positive for pluripotency markers, including TRA1-60, OCT3/4, NANOG and SOX-2 and were negative for SSEA1. Scale bars: 100 μm. Sample images of normal karyotypes analyzed by G- banding for the same iPSC lines shown next to immunocytochemistry images. (b) and (c) Pedigrees of family 1 and 2 respectively showing which individuals were cases (shaded) or controls (unshaded), individual diagnoses [ASD: autism spectrum disorder; DEP: depressive disorder; GAD: generalised anxiety disorder; ID: intellectual disability; PD-NOS: psychotic disorder (not otherwise specified); SCZ: schizophrenia], carrier status of the 16p13.11 microduplication (highlighted by *), and the clones that were derived from cases and controls (marked by blue arrows).

## Supplementary Figure 2.

Human iPSC demonstrate three germ layer differentiation. Pluripotency of all clones of iPSC were verified by differentiation into mesoderm (Brachyury and Eomes positive staining); endoderm (FOXA2, SOX17 and GATA-4 positive staining) and neuroectoderm (SOX1, PAX6 and NESTIN positive staining) germ layers. Scale bars: 100 μm.

## Supplementary Figure 3.

Neural conversion of iPSCs to cortical aNPCs. Proliferating aNPCs display characteristic rosette-like patterning in phase-contrast images (a) and uniform NESTIN expression and mosaic OTX2 expression (b). Phase-contrast images of uniform anterior cortical neuron cultures (c) which stain positive for Tuji, and MAP2 (d) with equivalent GFAP+ve astrocyte staining (<15%; mean astrocyte contamination of 6.7% and 8.0% for control and case lines respectively (e). cN’s generate action potentials upon depolarisation with current injection (f). Scale bar: 50 μm.

## Supplementary Figure 4.

Neuronal precursor cell lines from patients with 16p13.11 microduplication express elevated NDE1. iPSC were neuralised to NPCs (passage 8-10) and then cells were harvested to prepare, DNA, RNA or protein. (a) Schematic diagram showing the duplication region in the three cases together with the genes included in this region. We also carried out digital-drop PCR to verify that cases contained three copies of *NDE1* in the amplified region compared to two copies in the controls (not shown). (b) Following harvesting of RNA we performed RT-qPCR. *NTAN1, PDXDC1, NDE1* and *ABCC1* Taqman primer products were normalised to expression of three reference genes showing that cases express elevated levels of these 4 genes in the duplication compared to the controls. All data were derived from at least three independent derivations. (c) immunoblot of NPC total cell lysates probed with anti-

NDE1 polyclonal antibody and also with anti-vinculin loading control antibody. (d) quantification of NDE1 protein levels relative to loading control in pooled controls (3) compared to pooled cases (3). NPCs from cases expressed approximately 1.5 x the level of NDE1 protein compared to controls which is consistent with the patient lines expressing three copies of the NDE1 transcript.

## Supplementary Figure 5.

Human cerebral organoids recapitulate forebrain identity and show stereotypical organization and behaviour of progenitor cells. (a) Cerebral organoids formed large continuous neuroepithelia surrounding a fluid-filled cavity reminiscent of a ventricle with characteristic apical staining of the neural specific N-cadherin. (b) Staining with antibody to the forebrain marker FOXG1 demonstrates early brain regionalization in organoids at 1 month of age. (c) PAX6 staining shows successful neural induction and regions indicative of cerebral cortex formation at 1 month. (d) Organoids demonstrate dorsal cortical organization, organizing into a layer structure reminiscent of a ventricular zone with neurons localized to the basal surface. Mitotic radial glia staining (Phospho-Histone H3, green) in cortical regions of 1 month old cerebral organoids reveals inner radial glia (iRG) undergoing mitosis at the apical membrane (arrowheads) whereas outer radial glia (oRG) undergo mitosis outside the ventricular zone (arrows). Radial glia in the ventricular zone are marked by SOX2 staining (red). Approximately 85% of the PHis+ve cells are iRG cells and 15% are oRGs (+ 5.8%, *n*=10). (e) Organoids form the stereotyped layer structure of the mammalian cortex with CTIP2 stained neurons populating adjacent regions to the TBR1 stained pre- plate region. (f) NDE1 is expressed in the proliferative zones of cerebral organoids. NDE1 is most highly expressed in areas of high proliferation such as the VZ (similar to what is seen in mouse developing neocortex (Allen Developing Brain Atlas). (g) NF*κ*B p65 expression is localised to the apical surface of the ventricular zone in organoids as demonstrated by staining of 1-month old cerebral organoids with two different antibodies directed against NF*κ*B p65 (F6 is a mouse monoclonal to NF*κ*B p65 and C20 is a rabbit polyclonal to NF*κ*B p65). (h) Dual staining with anti-NDE1 and anti-NF*κ*B p65 antibodies [F6 and anti- phospho-NF*κ*B p65 (Ser536)] demonstrated co-localisation of staining to the VZ. Anti- phospho-NF*κ*B p65 (Ser536) also stains a number of nuclei of the VZ that also stain with anti-PH3 antibody (i).

## Supplementary Figure 6.

(a) Log2 transformed fold changes of the top 20 downregulated (green) and top 20 up- regulated (orange) genes in patient neural progenitors as compared to controls. Only ‘protein coding’ genes are listed which have a mean FKPM over all 12 samples>0.5. We selected the 20 most significant (by p-value) up-regulated and 20 most significant down- regulated protein coding genes which had a mean FPKM across all 12 samples > 0.5. These two sets of 20 genes were then ordered by decreasing magnitude of their log2 fold change. (b) Graph showing the p-value for enrichment via GO analysis of the top down- regulated GO term "Positive regulation of I-kappaB kinase/NF-kappaB signalling" (in blue) in differentially expressed genes above this cut-off versus all genes above this cut-off, and the p-value for enrichment of miR-484 targets (in red) in differentially expressed genes above this cut-off versus all genes above this cut-off p-values plotted as -log10(p). The horizontal black line indicates p=0.05 (i.e. values above this line are significant). While the GO term enrichment remains below p=0.05 as the FPKM cut-off increases, the miR-484 enrichment is already non-significant at a cut-off of FPKM > 0.5.

## Supplementary Figure 7.

Proliferation assays from each of the three individual cases studied. (a) Representative images of proliferation assays of all three case NPCs following treatment with vehicle (DMSO), TNF-*α*, compounds 1 and 2. (b) Graphs showing proliferation of each individual case NPC showing significant rescue of proliferation deficits following treatment with TNF-

*α* and compounds 1 and 2. (Ordinary one-way ANOVA with Dunnett’s multiple comparison test. Values plotted are the mean + SEM; *p = 0.05; **p < 0.005; ****p < 0.0001).
