# Supplementary material for: Reversal of proliferation deficits caused by chromosome 16p13.11 microduplication through targeting NFκB signaling: an integrated study of patient-derived neuronal precursor cells, cerebral organoids and in vivo brain imaging
Source: Mol Psychiatry. 2018 Nov 6;24(2):294–311. doi: 10.1038/s41380-018-0292-1 (PMC6344377; doi:10.1038/s41380-018-0292-1)
Supplement: Supplementary file 17 — Supplementary Methods [file 41380_2018_292_MOESM17_ESM.docx]

**Supplementary materials and methods**

*Clinical details of cases and controls*

Case 1 (highlighted in the pedigree shown in Supplementary Figure 1b) has a strong family history of major psychiatric disorder going back at least three generations and began to experience psychotic symptoms from 14 years of age. Two of the duplication carriers in this study were familial: a young man (case 3) with intellectual disability and ASD, diagnosed in his early teens with SCZ and his mother who had suffered from affective disorder for many years (case 2). Interestingly, an older sister of case 3 (daughter of case 2), is also a carrier of the 16p13.11 duplication, and has suffered from two prolonged episodes of puerperal psychosis (see pedigree shown in Supplementary Figure 1c). A further two older sisters of case 3 (also daughters of case 2), are unaffected, non-carriers, one of whom was also enrolled for stem cell studies (control 2). The Chr 16p13.11 duplication is located in a region with reported linkage to bipolar disorder^1^ and puerperal psychosis^2^. This family have a very strong family history of psychosis on the maternal side with two close cousins also diagnosed with schizophrenia. Psychiatric diagnosis according to DSM-IV(TR) criteria was established by consensus between two trained psychiatrists. Diagnostic information was obtained by a face-to-face semi-structured interview using the Structured Clinical Interview for DSM-IV (SCID) supplemented by reviews of hospital records and collateral information from hospital psychiatrists and General Practitioners. Controls 1 (Sc1901) and 2 (Sc1745) are the unaffected sister and father respectively of case 3^3^; controls 3 and 4 are unaffected non-carriers from the Scottish DISC1 t1:11 family where control 3 is ID No. 40 and control 4 is ID No. 28 in the published pedigree^4^; control 5 (line 34D6) was derived from a Caucasian, healthy female volunteer as previously described^5^.

*Analysis of MRI scanning data*

Three-dimensional high-resolution T_1_-weighted images of the entire brain (including the cerebellum and brainstem) with the A>>P phase encode direction were acquired using MPRAGE, with an approximate 3.44 x 3.44 x 5 mm voxel size, a 64 x 64 matrix, FOV of 220 x 220 mm, slice thickness of 5 mm, TE = 26 msec, TR = 1.56 sec, and a flip angle of 90°. Acquisition direction was top down. Imaging data was acquired at the Edinburgh QMRI imaging facility, at the University of Edinburgh. A detailed visual quality check of the regions were conducted according to standardised procedures^6^ with the purpose of excluding any outlier regions which were incorrectly parcellated. Formal N-of-1 statistical test methods have been developed for comparing an individual patient’s measurement to a control sample to test whether or not a patient exhibits a statistically significant difference^7^. This method provides a point estimate of the degree of abnormality of the individual measurement; i.e. it estimates the percentage of the control population that would obtain a lower/greater value than the patient. Formal proof that the *p* value for the significance test is also a point estimate of abnormality has been demonstrated^8^.

*Generation and characterization of human iPSC*

Plasmids incorporating Oct4/shP53, SOX2/Klf4 and L-Myc/Lin28 (pCXLE-hSK and pCXLE-hUL, Addgene plasmids 27077, 27078, 27080) were electroporated into dermal fibroblasts using an Amaxa Nucleofection Kit (Lonza). Colonies were selected between day 21-25 and individual colonies of cells were expanded into single wells coated with Geltrex (Life Technologies) in Essential 8 media (Life Technologies) at 37^o^C and 5% CO_2_. Prior to neuralisation iPSC were transitioned to reduced-growth factor Matrigel (BD Biosciences) coated plastic dishes. Quality control tests were performed after clonal passage 10 and included immunocytochemistry with a panel of antibodies to pluripotency markers (Tra-160, OCT3/4, NANOG, SOX-2). Standard G-banding chromosome analysis (karyotyping) was performed by the Cytogenetics Unit, at the Western General Hospital, Edinburgh. Episomal clearance PCR and microarray karyotype analysis (Affymetrix 750K Cytoscan array) were also performed. A cut-off of 5Mb is commonly used for CNVs in iPSC studies. Microarray was also used to confirm the presence/absence of the 16p13.11 duplication which is proposed to exert its effects from the result of dosage sensitive genes at this loci, in particular *NDE1*. We have shown by Affymetrix microarray and multiplex amplicon quantification (MAQ) PCR that all three patients carried a single copy gain of the short arm of chromosome 16 at band p13.11 where the duplication is 1.65 Mb in size spanning 14,892,975-16,544,033 (NCBI Build 17)^3^. Case 3 was also found, upon whole exome sequencing, undertaken as part of the UK10K project^9^, to carry a rare *de novo* loss-of-function mutation in *TSC2*.

*Three germ layer differentiation*

Glass coverslips (13-mm) were placed into wells of a 12-well plate and coated with poly-ornithine then Matrigel. Three wells were required to differentiate each iPSC line into each lineage (endoderm, mesoderm, neuroectoderm). iPSC were passaged and plated into these wells and allowed to reach approximately 30-50% confluency. To differentiate iPSCs into endoderm, each of three wells of iPSCs were cultured on day 1 into chemically defined media (CDM): 50% IMDM (ThermoFischer); 50% F-12+ GlutaMAX-1(ThermoFischer); 5 mg/ml BSA (Europa); 1% CD lipids (Invitrogen); 0.04 μl/ml 1-Thioglycerol (Sigma); 7 μg/ml Insulin (Roche); 15 μg/ml Transferrin (Roche) containing: 0.1 μg/ml Activin; 0.16 μg/ml FGF2; 0.01 μg/ml BMP-4; 10 μM Ly; 3 μM CHIR. On day 2 (approximately 24 hours later) the media was changed to CDM containing: 0.1μg/ml Activin; 0.16 μg/ml FGF2; 0.01 μg/ml BMP-4 and 10 μM Ly. Finally, on day 3 the media was changed to 1 ml/well of RPMI media containing: 0.1μg/ml Activin and 0.16μg/ml FGF2. Cells were fixed in 4% PFA and processed for immunocytochemical staining with FOXA2, GATA-4 and SOX-17 (see supplementary Table S2 for antibody details). To differentiate iPSCs into mesoderm, each of three wells of iPSCs were cultured on day 1 into CDM containing: 0.1 μg/ml Activin; 0.02 μg/ml FGF-2; 0.01μg/ml BMP-4; 10 μM Ly and 5 μM CHIR. After a further two days of differentiation and one more media change with same media, cells were fixed and stained for BRACHYURY and EOMES (Supplementary Table S2) To differentiate iPSCs into neuroectoderm, each of three wells of iPSCs were cultured on day 1 in CDM containing: 10 nM SB; 0.012 μg/ml FGF-2 and 0.15 μg/ml]/ml Noggin. Cells were grown for between 10-12 days with changes of media every two days and then fixed and stained with antibodies to SOX-1, PAX-6 and NESTIN (Supplementary Table S2).

*Generation of anterior NPCs (aNPCs)*

Human iPSCs were maintained on Matrigel (Corning), with Advanced DMEM/F12 (Advanced-DMEM/F12), 20% Knockout Serum Replacement, 10 ng/mL basic FGF-2, 1 mM L-glutamine, 100 mM 2-mercaptoethanol and 1% penicillin/streptomycin (P/S)(all media was purchased from Life Technologies). Human iPS cells were neurally converted in suspension in CDM as described^10, 11^. The media was changed to Base media (Advanced-DMEM/F12, 1% P/S, 1% Glutamax, 1% N-2), 0.4% B-27, 2.5 ng/mL FGF-2 upon observation of radially organised structures in neurospheres (10-21 days) and plated on Laminin (Sigma) coated tissue culture plates (Nunc) a week later. Neural rosettes were mechanically isolated, dissociated with Accutase (Sigma) and 20-40k cells were plated in one Laminin-coated well of a 96-well plate in proliferation media (Base media, 0.1% B-27, 10 ng/mL FGF-2 and 10 ng/mL EGF where stated). aNPCs were grown to high density before passaging 1:2 with Accutase on laminin coated plates until passage 5-6 and maintained on 1:100 Reduced-growth factor Matrigel-coated plates thereafter­­­­­ or cryopreserved as described^10^. All hiPSC and NPC lines were regularly checked and confirmed negative for mycoplasma. We demonstrated that our NPCs had the potential to differentiate into cortical neurons.

*Differentiation of aNPCs into cortical neuronal cultures*

aNPCs were plated in default media on poly-ornithine (Sigma), laminin (Sigma), fibronectin (Sigma) and Matrigel-coated coverslips for differentiation and fed twice a week. Default media was supplemented with 10 μM forskolin (Tocris) in weeks 2 and 3. From week 4 onwards forskolin was removed and default media was supplemented with 5 ng/mL BDNF and 5 ng/mL GDNF. Coverslips were then processed fixed and stained as previously described^11^.

*Immunohistochemical and immunocytochemical analysis*

Organoids were fixed in 4% PFA for 20 min at room temperature (RT) and then washed three times in PBS and cryoprotected in 30% sucrose in PBS overnight at 4^o^C. The tissues were embedded in 30% sucrose: OCT embedding media (VWR International) in plastic moulds and frozen by submersion in isopropanol on dry ice. Organoids were cryosectioned (15 μm thickness) as previously described^10^. For immunostaining, cells and organoids were permeabilised with 0.1% Triton X-100 in PBS (PBS-Tx), blocked for 4 hrs at room RT in 3% goat or donkey serum (Vector Labs) in PBS-Tx, followed by incubation with primary antibodies (Supplementary Table S2) overnight at 4^o^C. Secondary antibodies (Alexa Fluoro conjugated, Life Technologies) were diluted at 1:1000 in 3% goat serum in PBS-Tx for 2 hours at RT. Coverslips were mounted on slides with ProLong Gold antifade mountant with DAPI (ThermoFisher).

*Image acquisition and analysis*

Images were captured using a Nikon confocal microscope. Images were converted to TIFF format, corrected for brightness and contrast and analysed using ImageJ (NIH). All quantification was performed blind to the genotype and analysis was carried out by two independent blinded examiners. Files were allocated random numbering prior to image analysis and quantification. Figures for preparation were assembled using Adobe Illustrator (Adobe Inc.). Statistical analysis was carried out by first testing for normal distribution in which case ordinary one way ANOVA analysis was conducted. Where the data did not follow normal distribution then appropriate specific statistical testing was applied such as t-test (e.g. Figure 2b-unpaired two-tailed t-test with Welch’s correction; Figure 2f-paired t-test), and Mann-Whitney test (Figure 4i).

*RNA sequencing analysis*

QC analysis using an Agilent 2100 bioanalyzer demonstrated that all samples had RNA integrity number (RIN) values between 7.7-10. Total RNA-Sequencing was performed on the Illumina NextSeq550 platform utilising the TruSeq Stranded Total RNA with Ribo-Zero Gold kit at the Wellcome Trust Clinical Research Facility (WTCRF) at the Western General Hospital, Edinburgh. This protocol removed cytoplasmic rRNA and mitochondrial rRNA and produced libraries of approximately 150bp. Sequencing was carried out using high-output 150 cycle flow cells to provide approximately 400M 75bp paired-end reads (400M forward and 400M reverse reads). Samples were barcoded and ran in sets of 6 samples per flow cell ensuring at least 60M paired-end reads per sample. Raw reads in FASTQ format were mapped to the human reference genome (using sequence and annotations from Ensembl version 90^12^) using STAR version 2.5.3a^13^. Reads mapping to genes were then counted using featureCounts version 1.5.2^14^. Differential expression was performed using the DESeq2 R package, version 1.16.1^15^. Clones 2 and 3 for Case 1, and the two individual samples for Cases 2 and 3, were grouped together as the four samples of a "case" condition, while Controls 1-5 (3, 1, 2, 1 and 1 samples respectively) were grouped together as the 8 samples of a "control" condition. Fold changes and *p*-values for differential expression were then calculated for the comparison of "case" versus "control" conditions, this grouping of samples allowing the common gene expression changes among 16p13.11 microduplication patients to be extracted, while avoiding the reporting of expression changes particular to a single sample that are likely unrelated to the microduplication. GO enrichment analyses were performed using the topGO R package, version 2.28.0^16^. Sequence data has been deposited at the European Genome-phenome Archive (EGA), which is hosted by the EBI and the CRG, under accession number EGAS00001002895. Quantitative real-time PCR analysis and verification was performed on the same RNA samples as described below.

*Quantitative real-time PCR analysis*

RNA was extracted from cell pellets as described. cDNA was prepared using MMuLV reverse transcriptase and random hexamers (ThermoFisher) according to the manufacturer’s instructions. TaqMan gene specific primers were purchased from Life Technologies and used in a 96-well plate format to quantify differences in cDNA levels using a QuantStudio Flex Real-Time PCR machine (ThermoFisher) with TaqMan Universal PCR Master Mix (ThermoFisher). Technical replicates as well as no template and no RT negative controls were included and at least three biological replicates were studied in each case. The data were analysed using GeNorm analysis software and the qbase+ software (Biogazelle) for statistical comparisons. GeNorm analysis was performed on 15 samples and 10 reference genes (*ATP5B, TBP, TOP1, ERCC6, ENOX2, PRDM4, TYW1, RPLP0, HPRT1, UBC*) and the optimal number of reference targets in the experimental set-up was 3 (i.e. geNorm V<0.15 when comparing normalisation factor based on most stable reference targets which were *PRDM4 and TOP1*). ΔΔCt method was used to normalise and quantify relative fold changes in gene expression.

*RPPA sample preparation*

Nitrocellulose slide format RPPA was done according to published protocols^17^. Cell lysates for RPPA were prepared from NPCs grown to confluency on 6 well plates. Media was removed from the plates and wells were washed twice with PBS. Plates were placed on ice and all remaining PBS was aspirated off. Freshly prepared lysis buffer [1% Triton X-100, 50 mM HEPES, pH 7.4, 150 mM NaCl, 1.5 mM MgCl_2_, 1 mM EGTA, 100 mM NaF, 10 mM Na pyrophosphate, 1 mM Na_3_VO_4_, 10% glycerol, containing freshly added protease and phosphatase inhibitors (Roche Applied Science, Cat. 05056489001 and 04906837001, respectively)] was added to each well (75-100 μl/well) and incubated for 20 minutes on ice with shaking every 5 minutes. Cells were scraped off the plates and the cell lysate was collected into micro-centrifuge tubes and centrifuged at 14,000 rpm for 10 minutes at 4°C. The supernatant was aliquoted and stored at -80°C if required. Cellular protein concentration was determined by Bradford protein assay using Coomassie Plus Protein Assay (ThermoFisher) and BSA protein standard (2 mg/ml)(ThermoFisher). Absorbance was read in flat-bottomed 96 well plates with 595nm filter plate reader (FLUOstar Omega, BMG Labtech, Aylesbury, UK). The protein concentration was adjusted to 1 mg/ml with lysis buffer and the cell lysate was mixed with 4xSDS sample buffer without bromophenol blue (3 parts of cell lysate plus one part of 4xSDS sample buffer). Samples were heated to 80°C for 3 minutes and then printed as a concentration series in replicates and multiple exposure and curve fitting parameters were performed to ensure protein abundance measurements were conducted in the linear range to obtain a single value.

*Preparation of cellular extracts and immunoblotting*

Cytosolic and nuclear extract fractions were separated using a commercially available kit (NE-PER, Pierce) according to manufacturer’s instructions. Protein concentrations were determined using the Bradford protein assay (as above) and samples were aliquoted and stored at -80^o^C. Proteins (10-25 ug) were separated by NuPAGE Novex 4-12% Bis-Tris Midi Gels with MOPS Running Buffer (ThermoFisher) and transferred to PVDF membranes (Amersham Hybond P0.45, GE Healthcare) using a Bio-Rad Semi-Dry apparatus. Blocking was performed with 5% non-fat dried milk (Marvel) in TBS-T (20 mM Tris-HCL, pH 7.5, 137 mM NaCl, and 0.01% Tween) for 1 hr at room temperature. Membranes were incubated with primary antibodies (Supplementary Table S2) overnight at 4^o^C. Membranes were washed with TBS-T and incubated with horseradish peroxidase conjugated secondary antibodies (Dako) diluted at 1:10,000 in TBS-T containing 5% non-fat dry milk. Detection was performed using enhanced chemiluminescence reagent (ThermoFisher) according to manufacturer’s instructions. Blots were reprobed with loading antibody control (Abcam). Densitometric analysis was performed using ImageJ software (NIH). Experiments were repeated at least three times on three different cell preparations.

*Lentivirus production*

To generate the virus stocks, a 10 cm dish of HEK 293FT cells (ThermoFisher), grown to 80-90% confluency, were infected with 5 μg of each of viral envelope plasmid (pCMV-VSV-G), packaging plasmid (pCMVΔR8.91) and viral vector plasmid [pIRESpuro-RelA or pEGFP-N1 vector alone for NPC control infections (Clonetech)], using Lipofectamine 2000 (ThermoFisher). The pIRESpuro-RelA viral vector plasmid was generated by Professor Ron Hay (College of Life Sciences, University of Dundee) by inserting RelA (NFκB p65) cDNAs into the PIRESpuro-deNotI viral vector plasmid and gifted by Professor Neil Perkins (Newcastle University). Media, containing lentiviral particles, was harvested 48 hrs after transfection. The lentivirus containing media was centrifuged at 1200 rpm for 3 minutes and filtered through a 0.45 μm filter to remove cellular debris. The virus stock was aliquoted and stored at -80^o^C.

**Supplementary References**

1. McInnes LA, Escamilla MA, Service SK, Reus VI, Leon P, Silva S *et al.* A complete genome screen for genes predisposing to severe bipolar disorder in two Costa Rican pedigrees. *Proceedings of the National Academy of Sciences of the United States of America* 1996; **93**(23)**:** 13060-13065.

2. Jones I, Hamshere M, Nangle JM, Bennett P, Green E, Heron J *et al.* Bipolar affective puerperal psychosis: genome-wide significant evidence for linkage to chromosome 16. *The American journal of psychiatry* 2007; **164**(7)**:** 1099-1104.

3. Johnstone M, Maclean A, Heyrman L, Lenaerts AS, Nordin A, Nilsson LG *et al.* Copy Number Variations in DISC1 and DISC1-Interacting Partners in Major Mental Illness. *Molecular neuropsychiatry* 2015; **1**(3)**:** 175-190.

4. Thomson PA, Duff B, Blackwood DH, Romaniuk L, Watson A, Whalley HC *et al.* Balanced translocation linked to psychiatric disorder, glutamate, and cortical structure/function. *NPJ Schizophr* 2016; **2:** 16024.

5. Sullivan GJ, Hay DC, Park IH, Fletcher J, Hannoun Z, Payne CM *et al.* Generation of functional human hepatic endoderm from human induced pluripotent stem cells. *Hepatology* 2010; **51**(1)**:** 329-335.

6. Schmaal L, Veltman DJ, van Erp TG, Samann PG, Frodl T, Jahanshad N *et al.* Subcortical brain alterations in major depressive disorder: findings from the ENIGMA Major Depressive Disorder working group. *Mol Psychiatry* 2016; **21**(6)**:** 806-812.

7. Crawford JR, Howell DC. Regression equations in clinical neuropsychology: an evaluation of statistical methods for comparing predicted and obtained scores. *J Clin Exp Neuropsychol* 1998; **20**(5)**:** 755-762.

8. Crawford JR, Garthwaite PH. Methods of testing for a deficit in single-case studies: Evaluation of statistical power by Monte Carlo simulation. *Cogn Neuropsychol* 2006; **23**(6)**:** 877-904.

9. Singh T, Walters JTR, Johnstone M, Curtis D, Suvisaari J, Torniainen M *et al.* The contribution of rare variants to risk of schizophrenia in individuals with and without intellectual disability. *Nature genetics* 2017; **49**(8)**:** 1167-1173.

10. Stacpoole SR, Bilican B, Webber DJ, Luzhynskaya A, He XL, Compston A *et al.* Efficient derivation of NPCs, spinal motor neurons and midbrain dopaminergic neurons from hESCs at 3% oxygen. *Nature protocols* 2011; **6**(8)**:** 1229-1240.

11. Bilican B, Livesey MR, Haghi G, Qiu J, Burr K, Siller R *et al.* Physiological normoxia and absence of EGF is required for the long-term propagation of anterior neural precursors from human pluripotent cells. *PLoS One* 2014; **9**(1)**:** e85932.

12. Yates A, Akanni W, Amode MR, Barrell D, Billis K, Carvalho-Silva D *et al.* Ensembl 2016. *Nucleic acids research* 2016; **44**(D1)**:** D710-716.

13. Dobin A, Davis CA, Schlesinger F, Drenkow J, Zaleski C, Jha S *et al.* STAR: ultrafast universal RNA-seq aligner. *Bioinformatics* 2013; **29**(1)**:** 15-21.

14. Liao Y, Smyth GK, Shi W. featureCounts: an efficient general purpose program for assigning sequence reads to genomic features. *Bioinformatics* 2014; **30**(7)**:** 923-930.

15. Love MI, Huber W, Anders S. Moderated estimation of fold change and dispersion for RNA-seq data with DESeq2. *Genome biology* 2014; **15**(12)**:** 550.

16. Alexa A, Rahnenfuhrer J, Lengauer T. Improved scoring of functional groups from gene expression data by decorrelating GO graph structure. *Bioinformatics* 2006; **22**(13)**:** 1600-1607.

17. Macleod KG, Serrels B, Carragher NO. Reverse Phase Protein Arrays and Drug Discovery. *Methods Mol Biol* 2017; **1647:** 153-169.
